# Selecting lipopeptide‐producing, *Fusarium‐*suppressing *Bacillus* spp.: Metabolomic and genomic probing of *Bacillus velezensis* NWUMFkBS10.5

**DOI:** 10.1002/mbo3.742

**Published:** 2018-10-25

**Authors:** Adetomiwa Ayodele Adeniji, Oluwole Samuel Aremu, Olubukola Oluranti Babalola

**Affiliations:** ^1^ Department of Biological Sciences, Faculty of Natural and Agriculture Science North‐West University Mmabatho South Africa; ^2^ Food Security and Safety Niche Area, Faculty of Natural and Agriculture Science North‐West University Mmabatho South Africa; ^3^ Department of Chemistry, Faculty of Natural and Agriculture Science North‐West University Mmabatho South Africa

**Keywords:** *Bacillus velezensis*, ESI‐Micro‐Tof MS, genome, in silico, lipopeptides, plant disease

## Abstract

The results of this study indicate that the maize rhizosphere remains a reservoir for microbial strains with unique beneficial properties. The study sought to provide an indigenous *Bacillus* strain with a bioprotective potential to alleviate maize fusariosis in South Africa. We selected seven *Bacillus* isolates (MORWBS1.1, MARBS2.7, VERBS5.5, MOREBS6.3, MOLBS8.5, MOLBS8.6, and NWUMFkBS10.5) with biosuppressive effects against two maize fungal pathogens (*Fusarium graminearum* and *Fusarium culmorum*) based on 16S rDNA gene characterization and lipopeptide gene analysis. The PCR analysis revealed that lipopeptide genes encoding the synthesis of iturin, surfactin, and fengycin might be responsible for their antifungal activities. Few of the isolates also showed possible biosurfactant capability, and their susceptibility to known antibiotics is indicative of their eco‐friendly attributes. In addition, in silico genomic analysis of our best isolate (*Bacillus velezensis* NWUMFkBS10.5) and characterization of its active metabolite with FTIR, NMR, and ESI‐Micro‐Tof MS confirmed the presence of valuable genes clusters and metabolic pathways. The versatile genomic potential of our *Bacillus* isolate emphasizes the continued relevance of *Bacillus* spp. in biological management of plant diseases.

## 1 INTRODUCTION

In recent years, the focus has shifted from the use of environmentally harmful plant disease management practices such as the use of chemical pesticides and inorganic fertilizers to the development of ecological friendly approaches that do not pose a public health threat. The use of biological control agents (BCAs) in the management of cereal crop diseases has become popular (Babalola & Glick, 2012; Dimkić et al., 2013; Lugtenberg & Kamilova, 2009). Bacterial species from the genus *Bacillus* have gained prominent attention due to their tolerance to high temperature, ability to withstand adverse environmental conditions, ability to grow rapidly in liquid media and their ability to produce a large variety of secondary metabolites that have broad antimicrobial capabilities (Santoyo, Orozco‐Mosqueda, & Govindappa, 2012; Sumi, Yang, Yeo, & Hahm, 2014).

Members of the Gram‐positive endospore‐forming *Bacillus* sp., such as *B. amyloliquefaciens* and *B. subtilis* collected from plant parts, have been used in the control of fusariosis in small cereal grains including maize (Bacon & Hinton, 2011; Gond, Bergen, Torres, White, & Kharwar, 2015). Fusariosis in maize, which could manifest as Fusarium head blight (FHB) or Fusarium ear rot (FER) and many more, is caused by fusarium members such as *F. graminearum* and *F. verticillioides*. These diseases affect maize production in South Africa and other areas of the world (Boutigny et al., 2012, 2011 ; Summerell & Leslie, 2011). Maize, a staple crop in South Africa, is consumed daily in most households and used in the production of animal feeds (Janse van Rensburg, Mclaren, Flett, & Schoeman, 2015; Lamprecht, Tewoldemedhin, Botha, & Calitz, 2011); therefore, efforts to reduce loss due to preharvest and postharvest contamination by *F. graminearum* infection have recently gained significant attention (Boutigny et al., 2011; Mngqawa et al., 2016). The presence of mycotoxins zearalenone and deoxynivalenol found in maize grains infected by *F. graminearum* is also a cause for concern (Wang, Ndoye, Zhang, Li, & Liao, 2011).

Reports show that BCAs used for crop protection perform better in their native geographical regions due to increased survival rate compared to the use of imported commercial BCAs (Abiala, Odebode, Hsu, & Blackwood, 2015; Bardin et al., 2015; Grzywacz, Stevenson, Mushobozi, Belmain, & Wilson, 2014; Pereira, Nesci, Castillo, & Etcheverry, 2010). Our goal in this present work was to select indigenous *Bacillus* strains from the maize rhizosphere, evaluate their anti‐phytopathogenic potentials in vitro against *Fusarium* spp., molecularly characterize the *Bacillus* isolates, and identify the likely mechanisms they employ in their anti‐phytopathogenic activities. *Bacillus* spp. secrete lipopeptide compounds such as surfactin, fengycin, and iturin that they utilize in antibiosis. The presence of these cyclic lipopeptides in our maize root‐associated strains would be valuable if they are to be considered for *in planta* studies and subsequently for the management of *F. graminearum* infections in South Africa.

Strains within the genus have also been reported to synthesize structurally diverse secondary metabolites that exhibit broad‐spectrum antibiotic activities, and the genomic basis for the synthesis of these secondary metabolites has been attributed to the presence of polyketide synthases (PKSs) and non‐ribosomal peptide synthetase (NRPS) in their genomes (Raaijmakers, Bruijn, & Kock, 2006; Roongsawang, Washio, & Morikawa, 2011; Tyc, Song, Dickschat, Vos, & Garbeva, 2017). The amphipathic structure, the hydrophilic peptide portion, and a hydrophobic fatty acid portion of these peptides show resemblances. These peptides also exhibit a cyclic nature due to the linkage of their C‐terminal peptide residue either indirectly to a β‐hydroxy fatty acid or directly to a β‐amino acid (Mnif & Ghribi, 2015; Ongena & Jacques, 2008). These antimicrobial peptides have been isolated, quantified, purified, and characterized using various approaches and techniques that ensure the chemical components responsible for their bioactivity are well understood. The majority of the current approaches employed involve the combination of chromatographic techniques, mass spectrometry, nuclear magnetic resonance (NMR), and Fourier transform infrared spectroscopy (FTIR) (Biniarz, Łukaszewicz, & Janek, 2017; Jasim, Sreelakshmi, Mathew, & Radhakrishnan, 2016).

Reports have shown that expression of biosynthetic genes and secretion of secondary metabolites may be difficult during laboratory culture of potential BCAs due to growth conditions (Laureti et al., 2011). The non‐expression of genes or secretion of secondary metabolites can hinder the identification or detection of the specific metabolite or gene responsible for the antimicrobial activities of a BCA (Michelsen et al., 2015). To fully understand beneficial bacterial species, genomes of multiple independent isolates are required for comparison (Tettelin et al., 2005). Comparing the total repertoire of genes for a group of genomes from close bacterial species is an instrumental approach for the development of novel beneficial compounds and for the functional characterization of important genetic determinants in significant microbial strains (Medini, Donati, Tettelin, Masignani, & Rappuoli, 2005). The bacterial pan‐genome can be defined as the complete repository of genes located in the genome of closely related bacterial species. This includes the “core genome” (genes identified in two or more strains) and the “dispensable genome” (genes peculiar to single strains) (Medini et al., 2005; Rouli, Merhej, Fournier, & Raoult, 2015; Tettelin et al., 2005). The core and dispensable genes are crucial signatures for recognizing species diversity.

Researchers now use a combinational approach such as genome mining, pan‐genome analysis, structural data elucidation, and metabolomic characterization to identify biosynthetic products secreted by important microbes (Dunlap, Bowman, & Schisler, 2013; Van Der Voort et al., 2015). Often, genomic data offer predictions that lead to the detection of novel biosynthetic pathways, genes, and enzymes which then enables experimental isolation, structural elucidation, and chemical characterization of novel compounds (Challis, 2008). The combinational approach ensures that in silico or theoretically predicted biosynthetic products correlate with structurally or chemically identified metabolites (Ziemert, Alanjary, & Weber, 2016). Here, we examined the antimicrobial potential of the lyophilized extract of the secondary metabolites secreted by *Bacillus velezensis* NWUMFkBS10.5 while employing electro‐spray ionization mass spectrometry (ESI‐Q‐TOF MS), FTIR, and NMR to confirm that its secondary metabolites were functionally active.

Lastly, the genomic information of *B. velezensis* and other related *Bacillus* strains having strong potential for the control of phytopathogens including *F. graminearum* has been made available in recent years (Dunlap et al., 2013; Dunlap, Schisler, Bowman, & Rooney, 2015; Lee et al., 2015; Palazzini, Dunlap, Bowman, & Chulze, 2016; Pan, Li, & Hu, 2017). In light of this, we sequenced the genome of our best isolate NWUMFkBS10.5, to determine its phylogenomic association and its unique antimicrobial trait.

## 2 EXPERIMENTAL PROCEDURES

### 2.1 Description of sampling sites and sample collection from rhizosphere

Depending on the width of the maize plot and in no particular order, 20–30 g of rhizospheric soil was collected randomly from four maize rows, 15–25 m, apart in each plot from 10 maize farms in the North West Province of South Africa at harvest time. The geographic location of the sampling sites covers 28,206 km^2^ area. The temperature ranges between 17°C and 31°C during the summer and between 3°C and 21°C during the winter, with an average rainfall of 360 mm. Harvested maize plants were shaken gently at the roots to manually remove the loosely attached soil. The adhering root soil was considered as the rhizosphere soil, and these samples were pooled for each location. We obtained 10 different soil samples from the different maize plots.

### 2.2 Differential and selective isolation of *Bacillus* spp. from rhizosphere sample

Five grams of each soil sample was inoculated in 45 ml of LB (Sigma‐Aldrich L3522) broth and incubated for 16 hr with continuous shaking, at 150 *g* in an incubator at 35°C after which a calibrated inoculating loop was used to streak on the surface of 20 HiCrome™ (Sigma‐Aldrich) *Bacillus* commercial agar plates without polymyxin supplement and 10 *Bacillus* agar with polymyxin supplement (manufacturer protocols). HiCrome *Bacillus* agar is used for rapid identification of *Bacillus* spp. from a mixed culture by a chromogenic method. Following manufacturer's directions, distinct colonies were randomly selected from the agar plates of each sample. Grams reaction, oxidase activity, and catalase were performed to presumptively ascertain the genera of the selected isolates. Then, 200 isolates were selected and maintained at −80°C in Luria–Bertani (LB) broth with 15% (v/v) glycerol, and a 15 ml LB broth or agar slant of each isolate was kept at 4°C as working culture.

### 2.3 In vitro screening for *Fusarium*‐suppressing isolates

#### 2.3.1 Preliminary antagonistic activity

*Fusarium graminearum* and *Fusarium culmorum* were kindly provided by Dr Claire Prigent Combaret (UMR CNRS 5557) Microbial Ecology of Lyon, University Lyon 1, France, and Prof Cristina Cruz, Centre for Ecology, Evolution and Environmental Changes, Faculdade de Ciências da Universidade de Lisboa, Portugal, respectively, and they were maintained on potato dextrose agar (PDA Sigma‐Aldrich P2182) plates. Preliminary detection of the antagonistic activities of the 200 *Bacillus* isolates against *F. graminearum* was carried out by multiple confrontation dual culture tests. The protocols of Chen, Chen, Zhang, and Zhu (2014) were slightly modified. A 5‐mm‐diameter plug from an actively a growing (7‐day‐old) mycelial culture of *F. graminearum* was placed in the center of freshly prepared PDA plates (90 mm). From the 200 *Bacillus* isolates initially selected, six fresh colonies from 24 hr LB agar culture were circularly streaked (equidistance 1.5 cm) along each PDA plate at a distance of 1.5 cm from the edge of the plate using a sterile inoculating loop. Control plates consisted of *F. graminearum* placed on PDA alone. The plates were further incubated at 28°C for 7 days. Thereafter, only 11 isolates (BS1.1, BS2.7, BS3.5, BS4.3, BS4.6, BS5.5, BS6.2, BS6.3, BS8.5, BS8.6, and BS10.5) exhibiting strong inhibition were selected for further antifungal confirmatory tests.

#### 2.3.2 Confirmatory in vitro antifungal test

Plates were prepared as described above; however, antagonism was carried out against two fungal pathogens (*F. graminearum* and *F. culmorum*) in three conditions. (a) Single loop full of bacterial antagonist streaked at the center of PDA 3 days before both fungal agar plugs were inoculated on opposite sides (condition 1); (b) single loop full of bacterial antagonist streaked at the center of plate while simultaneously inoculating both fungal agar plugs on opposite sides (condition 2); and (c) single loop full of bacterial antagonist streaked 3 days after the fungal agar plugs were inoculated on opposite sides (condition 3). The *Bacillus* isolate with strong inhibition zones (around their streaks) against the two pathogens in the screening plates was selected for further characterization. The antagonistic effect was determined by measuring the zones of inhibition (mm). The percentage of growth inhibition was calculated using the formula.

$$\mathrm{PGI}\%=\left[\frac{C1-C2}{C1}\right]\times 100$$

where PGI is the percentage of growth inhibition, C1 is the control mycelia area of uninhibited fungi, and C2 is the distance between the bacterial colony and the growing edge of the fungal mycelia. Experiments were repeated three times, and the values were recorded as the means of three replicates.

### 2.4 Detection of biosurfactant ability

#### 2.4.1 Hemolysis blood agar test

Isolates, BS1.1, BS4.6, BS5.5, BS6.3, BS8.5, BS8.6, and BS10.5, were subjected first to hemolysis test, as isolates with biosurfactant‐producing capability can lyse erythrocytes. A colony loopful of fresh cultures of each isolate or 20 μl of each fresh culture in LB broth was taken and streaked on blood agar plates (HiMedia, India). Plates were incubated from 48 to 72 hr at 37°C (Chakraborty, Chakrabarti, & Das, 2014). The plates were observed for clear zones around the colonies. However, a clearing zone around the bacterial colony on blood agar is not always confirmatory for biosurfactant production (Hazra et al., 2011; Youssef et al., 2004).

#### 2.4.2 Drop collapse test and microplate assay

To determine the production of biosurfactant compounds, a modified “drop collapse test,” applied according to Yanes, Fuente, Altier, and Arias (2012), was conducted. Briefly, each well of a 96‐well plate lid coated with 2 μl of commercial test substances consisting of vegetable oil, motor engine oil, kerosene, hexadecane, and paraffin oil was equilibrated for 2 hr. A cell‐free supernatant from an overnight LB broth culture of each isolate was prepared by filtering through 0.22 μm nitrocellulose membranes (Millipore Corporation, Bedford, MA, USA), and a 5 μl drop of the cell‐free supernatant of each isolate was then placed in the center of the coated well. The result was determined visually after 1 min. If the drop remained beaded, the result was scored as negative, and if the drop collapsed, the result was scored as positive. Water and SDS were used as negative controls of the media. Each treatment was repeated three times. Drop collapse test and microplate assay were also carried out according to the method described by Ben Belgacem et al. (2015). For the microplate assay, 100 μl supernatant was pipetted into a well of 96 microplate and the plate was viewed using a backing sheet of white paper with black grid. A positive result is indicated by the surfactant causing some wetting at the edge of the well and the fluid taking the shape of a diverging lens. The negative result is based on the premise that pure water in a hydrophobic well shows a flat surface, or an optical distortion is observed when surfactants are added to an aqueous solution.

### 2.5 Extraction of genomic DNA

We further chose seven isolates (BS1.1, BS4.6, BS5.5, BS6.3, BS8.5, BS8.6, and BS10.5) out of the 11 *Bacillus* isolates, based on their strong antifungal activity, for molecular analysis. Genomic DNA was extracted from overnight culture of the selected isolates using the Zymo Research ZR Soil Microbe DNA Miniprep genomic isolation kit (Epigenetics) following manufacturer's procedure. DNA quantity and quality were assessed with spectroscopic methods using a NanoDrop 1000 (Thermo Scientific, Wilmington, DE, USA), and the DNA was used as the template for polymerase chain reaction (PCR) analysis.

### 2.6 Detection of lipopeptide genes and molecular characterization of *Bacillus* isolates

Identification of the selected *Bacillus* isolates was by 16S rDNA gene sequencing (Garbeva, Veen, & Elsas, 2003), and the presence of lipopeptide genes in the DNA extracts of the *Bacillus* isolates was determined with a 25 μl reaction mixture containing 1.5–2.5 μg of template DNA; 1 μl of primer, 12.5 μl OneTaq Quick‐Load 2× master mix with standard buffer (New England Biolabs NEB), and 9.5–10.5 μl nuclease‐free water in PCR thermocycler. All the primers utilized in PCR amplification protocols were synthesized by Whitehead Scientific, Integrated DNA Technologies (Supporting Information Table S3). The PCR amplicons were analyzed by electrophoresis in 1% (w/v) agarose gel, and the sizes of the bands were determined using 1‐kb molecular marker. The gel containing 10 μg/ml ethidium bromide (Bio‐Rad) was visualized using a gel documentation system (Gel Doc 2000, Bio‐Rad) to confirm the expected size of the PCR products. NucleoSpin Microbial DNA Purification Kit (Macherey‐Nagel) was used to purify the PCR products which were then sent to Inqaba Biotec (Pretoria, South Africa) for sequencing. 16S rDNA sequences were blast searched on the NCBI GenBank and ENA database (default settings). Aligned sequences were analyzed using MEGA 7.0 software (Tamura et al., 2011), and phylogenetic trees were reconstructed based on the 16S rDNA gene using the neighbor‐joining methods (Saitou & Nei, 1987). Topological robustness was evaluated by bootstrap analysis (Felsenstein, 1985) based on 1,000 replicates.

### 2.7 Extraction, collection of cell‐free supernatant, and purification of secondary metabolites

From the seven antagonistic subsets, isolate BS10.5 (NWUMFkBS10.5) was the most effective with the highest number of encoding genes, and it was chosen for further analysis. The production and purification of BS10.5 active metabolites were done according to Gond, Bergen, Torres, and White (2015) with slight modification. Cell‐free antimicrobial substances from BS10.5 were collected after the rhizobacteria were grown in 1 L LB broth at 30°C with continuous shaking at 200 *g* for 72 hr. The cells were harvested by centrifugation at 13,000 *g* for 15 min, and the culture supernatant was filter sterilized through 0.22 µm nitrocellulose membranes (Millipore Corporation) filters to obtain cell‐free supernatants. About 100 ml of cell‐free supernatant was stored for anti‐pathogen test. Isolate BS10.5 was further grown in 1 L LB broth at 30°C with constant shaking at 200 *g* for 4 days. After fermentation, the cell filtrate was collected by centrifugation at 6,000 *g* for 15 min at 4°C, and the supernatant was acid‐precipitated by adjusting to pH 2.0 with 6 M HCl. After an overnight incubation at 4°C, the precipitate was centrifuged at 8,000 *g* at 4°C for 15 min and the pellet was dissolved in methanol–water (50:50) and then filtered through 0.22 µm PTFE membrane filter to remove larger particles and cell components. The mixture was then concentrated by vacuum evaporator at 45°C and then finally lyophilized.

### 2.8 Effect of culture‐free supernatant and lyophilized extracts of isolate BS10.5 on bacterial pathogens and *Fusarium* pathogens

#### 2.8.1 Antibacterial activity

The activity of the cell‐free supernatants was determined by disk diffusion assay. Sterile filter paper disks were impregnated with 60 µl of the cell‐free supernatant of BS10.5, and the disks were placed on a MHA plate previously inoculated with bacterial pathogens (BC = *Bacillus cereus* ATCC 10876, EF = *Enterococcus faecalis* ATCC 29212, KP = *Klebsiella pneumoniae* ATCC 25923, and PA = *Pseudomonas aeruginosa* ATCC 27853). Control plates included antibiotic disks of norfloxacin (5 μg/disk) and tetracycline (30 μg/disk). The plates were incubated overnight at 25°C and 28°C. Antibacterial activity was observed as inhibition zones around the disk, and the experiment was conducted twice in triplicates.

#### 2.8.2 Antifungal activity

Sterile filter paper disks impregnated with 60 µl of the cell‐free supernatant of BS10.5 were placed at the far edge of a PDA plate, and 5‐mm agar plugs of the *Fusarium* pathogens (*F. graminearum* and *F. culmorum*) were transferred to the opposite edge of the PDA plates. Nystatin (30 μg/disk) was used as control, and after 7 days of incubation and observation of plates at 25°C, zones of inhibition were recorded.

### 2.9 Dose‐dependent anti‐pathogenic activity of the BS10.5 lyophilized extract

Disk diffusion was employed for the antimicrobial assay following the method described by Chen, Wang, Wang, Hu, and Wang (2010) with modifications. The lyophilized extract was dissolved in phosphate‐buffered saline (PBS) (pH 7.5) to a concentration of 10 mg/ml and serially diluted to varying concentrations of 100, 90, 70, 50, and 30 µg/ml. Sterile disks made from punctured filter papers were then impregnated with 40 µl of the solutions and allowed to dry. Impregnated disks were then placed on the periphery of freshly prepared PDA plate containing a 5‐mm disk of the fungal pathogens in the center or a MHA plate in which 100 µl of overnight cultures of the bacterial pathogens at OD 0.5:600 nm had been spread. Forty microliters of PBS (pH 7.5) was used as a control disk. Antimicrobial activity was observed by the inhibition of microbial growth around the disk. The plates were incubated for 5 days at 28°C for fungal pathogens and 2 days at 28°C for bacterial pathogens (KP, PA, MC, and EF). Each test consisted of three replicates. Recordings were taken if zones of inhibition were spotted from the border of the disk to the perimeter of visible pathogen.

### 2.10 Effect of the BS10.5 lyophilized extract and commercial fungicides on fungal mycelia growth

Using well diffusion, the activity of the BS10.5 extract powder was also compared to the activity of commercial fungicides using PBS as diluent following a modified protocol of Mousa, Shearer, Limay‐Rios, Zhou, and Raizada (2015). 0.2 g/ml of the lyophilized extract, 10 µg/ml of amphotericin B, and 10 µg/ml of nystatin and triazole (10 µg/ml) were prepared with PBS (pH 7.5). Each mixture was then loaded into each of the wells (made previously with bottom parts of 200‐µl sterile pipette tips) of PDA plates previously inoculated with a 5‐mm agar plug of fungal pathogens. The plates were incubated for 5 days at 28°C, and each plate consisted of three replicates. Recordings were taken if zones of inhibition were spotted from the border of the well to the perimeter of visible pathogen.

### 2.11 Identification and characterization of BS10.5 bioactive compounds by NMR, FTIR, and ESI‐Q‐TOF MS analysis

#### 2.11.1 Fourier transform infrared spectroscopy

Fourier transform infrared (FTIR) spectroscopy identifies the types of chemical bonds and functional groups in compounds, and it is useful in elucidating the components of an unknown sample. Ten microgram of the lyophilized extract of BS10.5 was analyzed with transform alpha (FTIR) KBr‐integrated spectrometer (Bruker). Spectrum reading was at 400–4,000 wavenumbers (cm^−1^) with an average of 32 scans. Spectrum was viewed and collated with the OPUS spectroscopy software.

#### 2.11.2 Nuclear magnetic resonance spectroscopy (NMR)

Twenty microgram of the lyophilized extract of BS10.5 was dissolved in 0.5 ml of deuterated DMSO (dimethyl sulfoxide), and ^1^H and ^13^C NMR spectrum were acquired on the specific signal assignment. NMR spectra were recorded using a Bruker Avance III 500 MHz spectrometer at room temperature with chemical shifts () recorded against the internal standard tetramethylsilane (TMS).

#### 2.11.3 Mass spectrometry analysis by ESI‐Q‐TOF MS

A high‐resolution mass spectrum was obtained for the lyophilized extract of BS10.5 with an Applied Biosystems 4800 Plus MICRO‐TOF/TOF analyzer (AB Sciex, USA) operated in the positive ion mode with an accelerating voltage of 20 kV, 337 nm nitrogen laser for ionization and α‐cyano‐4‐hydroxycinnamic acid for matrix. Bruker compass data analysis was used to process the mass spectrometry data while molecular weights and formulae were characterized by mass spectrum smart formula tools. The FTIR, NMR, and mass spectrometry analyses were carried out at the Chemical Resource Beneficiation/Laboratory Analytical Services of the North‐West University (Potchefstroom, South Africa).

### 2.12 Genome sequence of isolate NWUMFkBS10.5

The whole genome of the *Bacillus* strain NWUMFkBS10.5, (the preferred isolate), was extracted using the Zymo Research ZR Soil Microbe DNA Miniprep Genomic Isolation Kit and sequenced with Illumina MiSeq Reagent Kit v2 microsystem by MR DNA (Shallowater, TX, USA) using their protocols. The Kbase (Arkin et al., 2016) platform was used to check quality of reads (FastQC v.1.0.1)**,** trim reads (Trimmomatic v0.32), and close gaps and remove adaptor sequences (Cutadapt v1.0.1). The reads were also assembled into contigs on the Kbase platform using ARAST v0.0.4 (Velvet and Kb_SPAdes v0.0.9). Contigs from the Kbase were uploaded on the RAST Server version (v) 2.0 (Aziz et al., 2008), PATRICK v3.3.15 (Wattam et al., 2017), and NCBI Prokaryotic Genome Automatic Annotation Pipeline (PGAAP v4.2) (Pruitt, Tatusova, Brown, & Maglott, 2011) for automated annotation. Default parameters were used for the bioinformatics analysis.

### 2.13 Data mining and in silico analysis of NWUMFkBS10.5 genome

Anti‐smash (4.0.0rc1) and PRISM were used to predict the biosynthetic products and genetic clusters present in BS10.5. A Pan‐genome was created on the Kbase platform to run a comparison of the total genes present in BS10.5 and other established biocontrol *Bacillus* strains.

### 2.14 Statistical analysis

A multivariate general linear model was used to analyze treatment means and inhibition rates. Least significant difference test (LSD), Duncan multiple test, and Student–Newman–Keuls (SNK) test were used to compare observed means, pathogen–antagonist relationship, treatment effects, and effect of conditions of inoculation using SPSS statistical software (version 22) at the significance level of 5%.

## 3 RESULTS

### 3.1 Presumptive selection and identification of bacterial isolates

**Figure 1:**
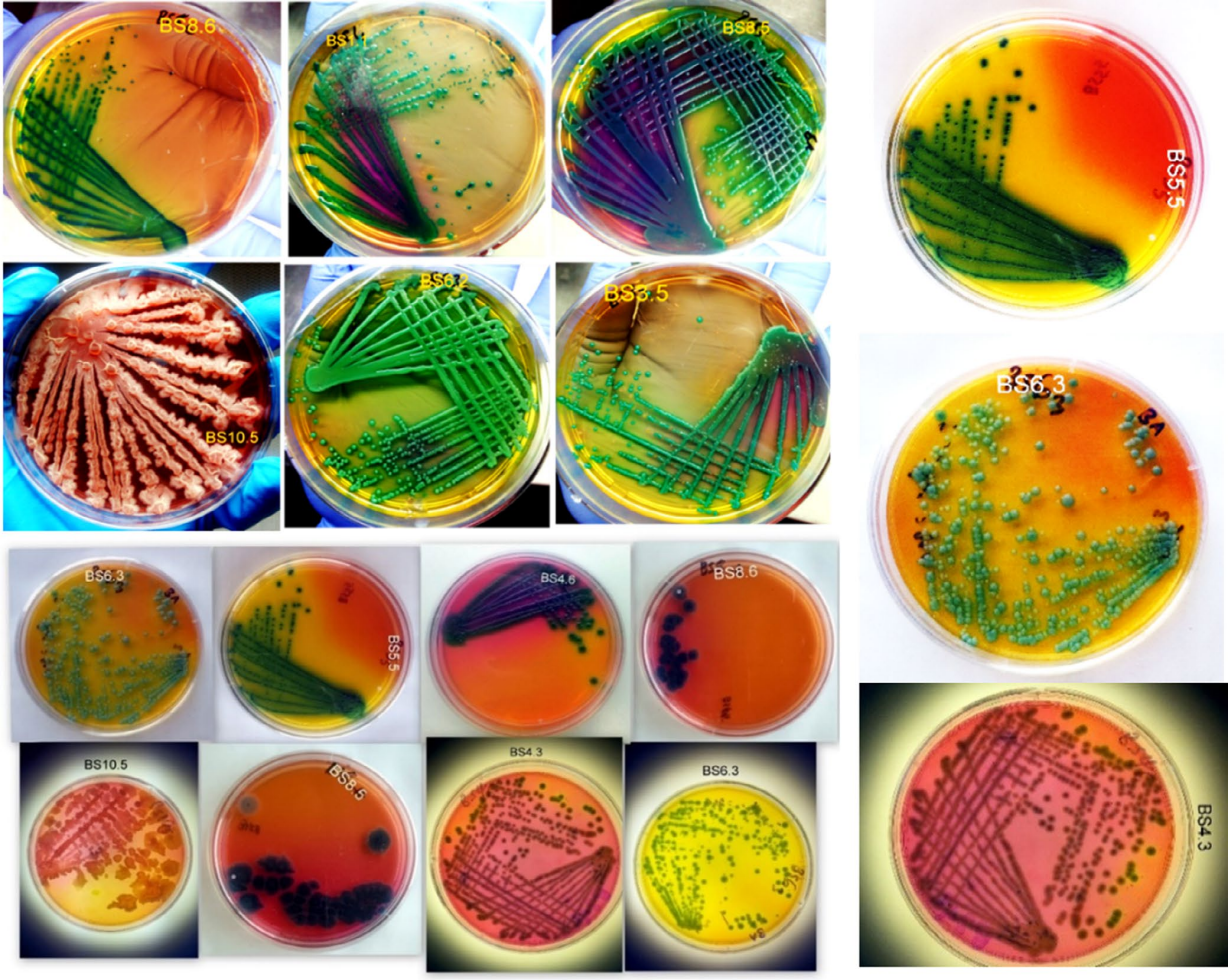
Colonial characteristics and presumptive identification of the isolated *Bacillus* strains on HiChrome Bacillus agar

All the 200 isolates selected from the HiChrome *Bacillus* agar according to manufacturer's descriptions (Supporting Information Table S1) were Gram‐positive, oxidase‐positive, and catalase‐positive. Supporting Information Table S2 further describes the location and total number of isolates selected from each site. A large percentage of the isolates showed cultural characteristics that resembled *B. subtilis* strains because of their light green to green colonies on the agar. Isolate BS10.5 showed a unique cultural characteristic that was not included in manufacturer's identification profile (Figure 1), so, as such, it was not assigned to any of the *Bacillus* specie. Isolates BS8.5 and BS8.6 showed characteristics resembling strains of *B. thuringensis*. Isolates BS4.6 and BS4.3 had close resemblance with *B. cereus* while BS6.3 shared a close resemblance with *B. pumilis*. Characteristics that resembled *B. coagulans* were seen in isolates BS1.7, BS2.7, and BS3.5.

### 3.2 *Bacillus* inhibition of *Fusarium* mycelia

Antagonistic activity of the *Bacillus* isolates against the two *Fusarium* pathogens showed that lower inhibition rates were seen in condition 1 (antagonist inoculated on PDA 3 days before pathogens) and condition 2 compared to condition 3, where the fungal mycelia plug was inoculated on the PDA 3 days before the antagonists were inoculated (Tables 1 and 2). Overall, *F. culmorum* was more resistant to the antagonists during condition 1 and condition 2 when compared with susceptibility of *F. graminearum* during those conditions of treatment. However, the inhibition of *F. graminearum* was less during condition 3.

**Table 1:** Percentage inhibition of *Fusarium graminearum* mycelia by *Bacillus* isolates

Treatment	Condition (% inhibition zone)			Mean
	1	2	3	
BS1.1	42.67	46.67	66.67	52.00b
BS2.7	42.67	41.33	56.00	46.67cd
BS3.5	45.33	41.33	68.00	51.56b
BS4.3	40.00	38.67	44.00	40.89f
BS4.6	42.67	38.67	44.00	41.78ef
BS5.5	36.00	30.67	58.67	41.78ef
BS6.2	32.00	26.67	69.33	42.67ef
BS6.3	36.00	33.33	65.33	44.89de
BS8.5	45.33	46.67	54.67	48.89bc
BS8.6	40.00	37.33	70.67	49.33bc
BS10.5	54.67	42.67	77.33	58.22a
Mean	41.58b	38.55c	61.33a	
ANOVA				
Treatment (T)	***			
Condition (C)	***			
T × C	***			

Note

Values are means and standard error of four replicates of in vitro antagonistic activity of selected *Bacillus* isolates against *F. graminearum*; values having the same letters are not significantly different according to Duncan's least significant difference test at *p* ≤ 0.05. 1, 2, and 3 represent conditions of inoculation. ^***^ = treatment, conditions and treatment and conditions are significantly different.

**Table 2:** Percentage inhibition of *Fusarium culmorum* mycelia by *Bacillus* isolates

Treatment	Condition (% inhibition zone)			Mean
	1	2	3	
BS1.1	37.33	34.67	60.00	44.00e
BS2.7	44.00	41.33	62.67	49.33d
BS3.5	45.33	46.67	57.33	49.78cd
BS4.3	45.33	50.67	70.67	55.56ab
BS4.6	53.33	45.33	53.33	50.67cd
BS5.5	45.33	44.00	65.33	51.56cd
BS6.2	37.33	36.00	46.67	40.00f
BS6.3	56.00	50.67	69.33	58.67a
BS8.5	52.00	54.67	62.67	56.44a
BS8.6	50.67	57.33	50.67	52.89bc
BS10.5	45.33	38.67	70.67	51.56c
Mean	46.55b	45.45b	60.85a	
ANOVA				
Treatment (T)	***			
Condition (C)	***			
T × C	***			

Note

Values are means and standard error of four replicates of in vitro antagonistic activity of selected *Bacillus* isolates against *F. culmorum*; values same having the same letters are not significantly different according to Duncan's least significant difference test at *p* ≤ 0.05. 1, 2, and 3 represent conditions of inoculation. ^***^ = treatment, conditions and treatment and conditions are significant different.

### 3.3 Detection of biosurfactant production

The multiple test on the seven isolates for detecting biosurfactant production revealed that four of the isolates (BS1.1, BS3.5, BS8.6, and BS10.5) are biosurfactant producers (Table 3). The detection of biosurfactant production in BS1.1, BS3.5, BS8.6, and BS10.5 further correlates the PCR detection of surfactin genes in these isolates, which is not the case with isolates BS5.5, BS6.3, and BS8.5. The stability of the biosurfactants produced under different growth conditions, however, might need to be determined because the production of biosurfactants is dependent on conducive pH, temperature, and salinity concentration (Rivardo, Turner, Allegrone, Ceri, & Martinotti, 2009).

**Table 3:** Test for biosurfactant properties of potential isolates

Isolates	Test substance						
	Drop collapse						
	Blood agar^a^	Microplate assay	Vegetable oil^b^	Motor engine oil^b^	Kerosene^b^	Hexadecane^b^	Parafilm^b^
BS1.1	●	●●	●	●	●	●	●
BS3.5	●	○	●	●	●	○	●
BS5.5	●	○	○	○	●	○	○
BS6.3	●	○	○	○	○	○	○
BS8.5	●	○	○	○	○	○	○
BS8.6	●	●●	○	●	●	●	●
BS10.5	●	●●	●	●	●	●	●
Water	○	○	○	○	○	○	○
SDS	●	●●	●	●	●	●	●

Note

a = hemolysis test: b‐hemolysis (●), no hemolysis (○); b = Drop collapse assay ●: collapse; ○: no collapse; hemolysis test ●: positive; ○: negative; drop collapse test: negative (○), spreading (●), comparable with sds (●); microplate assay: optical distortion comparable with water (○), optical distortion comparable with sds (●●).

### 3.4 Molecular characterization of *Bacillus* isolates

The PCR analysis carried out against the seven isolates revealed that each isolates harbored at least one lipopeptide gene. PCR amplification was carried out according to previously established protocols. The targeted genes and primers utilized during the PCR amplification are shown in Supporting Information Table S3. Multiple lipopeptide antibiotic genes were detected in BS10.5 amplicon (Table 4), and all the isolates showed a positive result for the fengycin (Af2‐F) primer. Clusters for surfactin genes were detected in all the isolates using the *SrfC* primer. However, only three isolates harbored iturin genes, and no amplification was detected using the fengycin (*FenD*), *Ipdc* (indole pyruvate decarboxylase), and *Acc* deaminase primers. Single predominant amplicon bands were produced from the PCR products from the gel electrophoresis (Supporting Information Figure S1). The presence of the detected lipopeptide antibiotics could be responsible for the antagonistic activities of the *Bacillus* isolates against fungal pathogens.

**Table 4:** Genes detected in the antagonistic *Bacillus* isolates using specific primers sets

Primer set	Isolates						
	BS10.5	BS8.6	BS8.5	BS6.3	BS5.5	BS4.6	BS1.1
Iturin A (*ItuD*)	●	○	○	●	○	●	○
16S (*BacF*/R1378)	●	●	●	●	●	●	●
Surfactins (As1‐F)	●	●	●	○	●	○	●
Fengycins (Af2‐F)	●	●	●	●	●	●	●
Surfactin (*SrfC*)	●	●	●	●	●	●	●
Surfactin (*sfp*)	●	○	○	○	○	○	○
Fengycin (*FenD*)	○	○	○	○	○	○	○
Bacillomycin D (*BamC*)	●	○	○	○	○	○	○
*Ipdc* (Indole Pyruvate decarboxylase)	○	○	○	○	○	○	○
*Acc* Deaminase	○	○	○	○	○	○	○

Note

●: (Positive) a PCR amplicon of expected size was seen; ○: negative.

### 3.5 Phylogenetic analysis

Based on the blast search of the partial 16S rDNA gene sequences and submission to NCBI, GenBank accession numbers were assigned to the seven isolates (Supporting Information Table S4). The blast search correlated with the presumptive identification from the chromogenic agar. Using the aligned MAFFT 16S rDNA sequences, the phylogenetic tree was constructed with MEGA 7.0 software. In addition, the bootstrap support of the genetic relatedness among six of the isolates (BS1.1, BS4.6, BS5.5, BS6.3, BS8.5, BS8.6, and BS10.5) and their nearest neighbors was 100% except isolate BS5.5 that showed 71% close match. In addition, isolates BS1.1, BS5.5, and BS6.3 had no direct clustering with any strain on the tree (Supporting Information Figure S2).

### 3.6 Antimicrobial activity of cell‐free supernatants

The results of the antimicrobial activity of the cell‐free supernatants showed that the cell‐free culture filtrate from BS10.5 contains strong bioactive substances with inhibitory potentials against fungal and bacterial pathogens (Supporting Information Table S5). The cell‐free filtrates inhibited *F. graminearum* and *B. cereus* at the same rate, however, but inhibited *K. pneumoniae* and *P. aeruginosa* moderately. In comparison with antibiotics used against the fungal pathogens, the cell‐free supernatant exhibited an inhibition level close to that of nystatin. However, it showed higher inhibitory potential than tetracycline and ciprofloxacin against the bacterial pathogens based on the concentration of the antibiotics used.

### 3.7 Antipathogenic activity of the BS10.5 lyophilized extract at different concentrations

**Figure 2:**
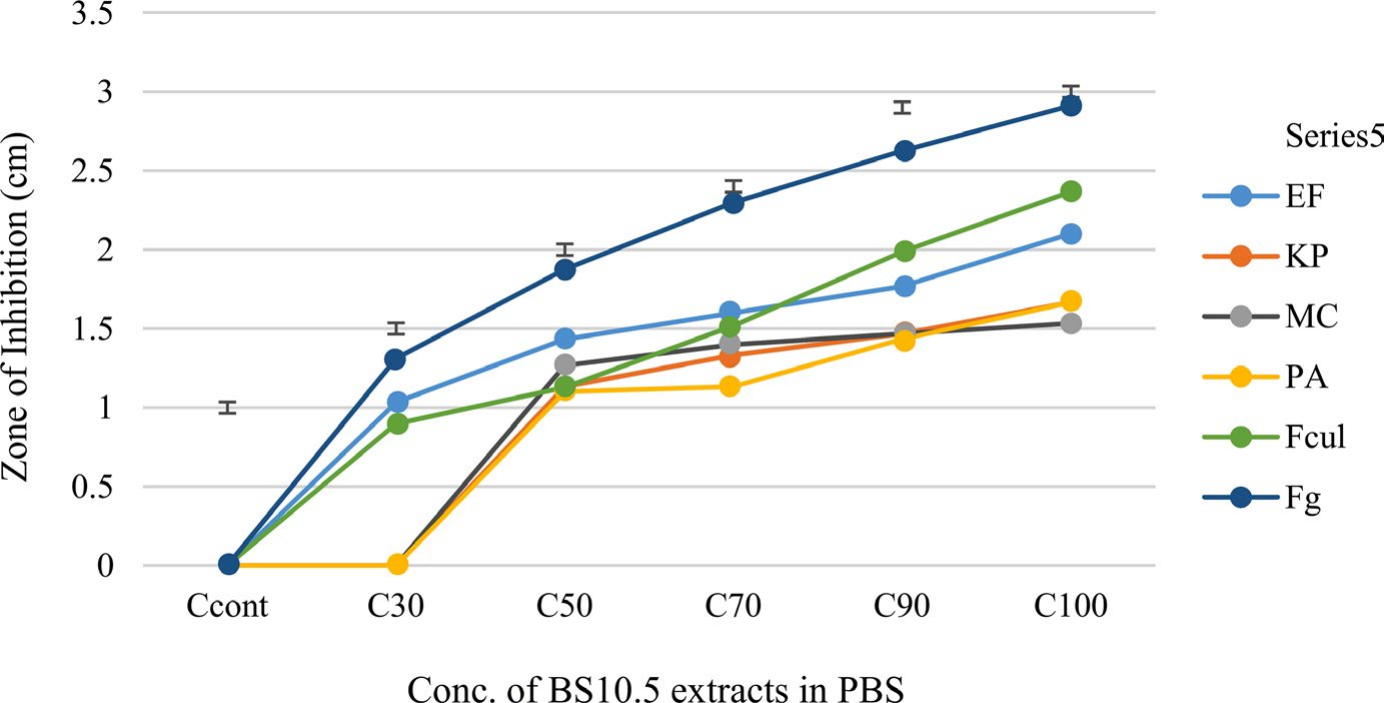
Means of three replicates showing activity of BS10.5 extracts on the microbial pathogens at different concentrations. Values are significantly different according to Duncan's least significant difference test at *p* ≤ 0.05, and means are significantly different from the control

During the lyophilized extract antipathogenic test, the effect of the extracts decreased relative to an increase in dilution with PBS. Among the bacterial pathogens, EF was the most susceptible to the lipopeptides extracts of BS10.5, while MC was the least susceptible. *F. graminearum* was more sensitive to the extract concentrations than *F. culmorum* (Figure 2). At 30 µl, three of the pathogens (PA, MC, and KP) showed no sensitivity.

### 3.8 Comparative antifungal activity of the BS10.5 lyophilized extract and commercial fungicides

**Figure 3:**
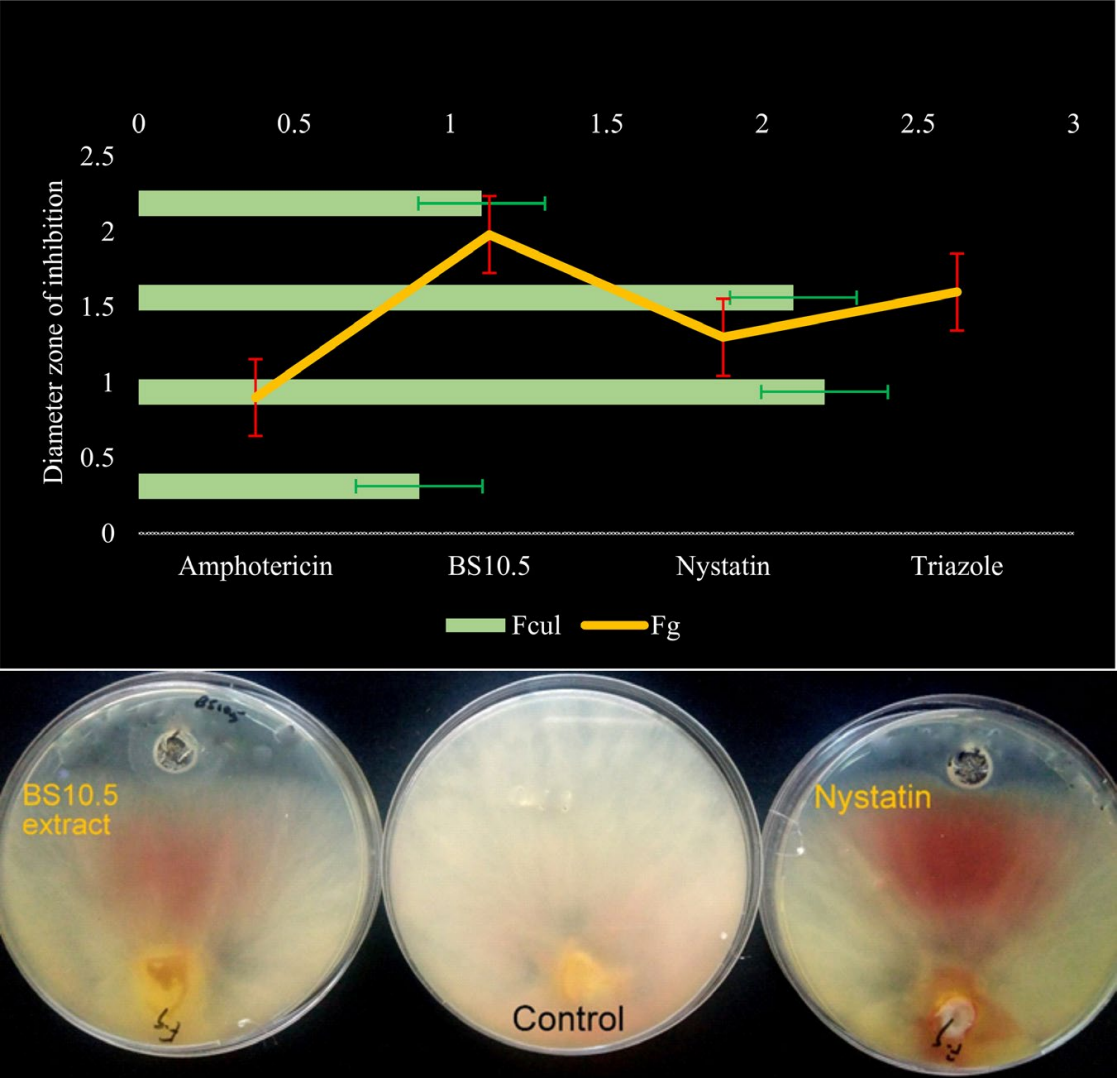
Upper plate: inhibitory effect of the BS10.5 extract (20 mg/ml) and fungicide controls (triazole, amphotericin B, and nystatin) (at concentrations 10 µg/ml, respectively), on the *Fusarium graminearum* and *Fusarium culmorum* growth in vitro; Lower plate: inhibitory effect of the BS10.5 extract (20 mg/ml) and nystatin (10 µg/ml), on the *F. graminearum*. For the experiment, *n* = 4. Activity of the lyophilized extract and commercial fungicide on fungal growth were significantly different at *p* ≤ 0.05

The BS10.5 extract showed a higher inhibition rate against *F. graminearum* than the commercial fungicides (nystatin and amphotericin) (Figure 3).

### 3.9 FTIR, NMR, and ESI‐Q‐TOF MS analysis of the bioactive compounds present in extracts of BS10.5

#### 3.9.1 FTIR analysis

The FTIR absorbance showed functional groups corresponding to OH at 3,600–3,500 cm^−1^, CH stretch at 3,000–2,500 cm^−1^, NH stretch at 2,500–2,000 cm^−1^, COO‐ at 1,900–1,500 cm^−1^, and CC and CN at 1,500–1,000 cm^−1^ (Supporting Information Figure S3). These wave numbers show characteristics similar to lipopeptides. The stretching and vibration mode of the absorbance is indicative of aliphatic chains, alkyl chains, peptide bonds, and two amide bonds, signifying the presence of a compound with ester and amino groups. This result, which is consistent with the report of Romero et al. (2007), Nam et al. (2015), Rivardo et al. (2009), is indicative of fengycin, surfactin, and iturin moieties.

#### 3.9.2 NMR analysis

**Figure 4:**
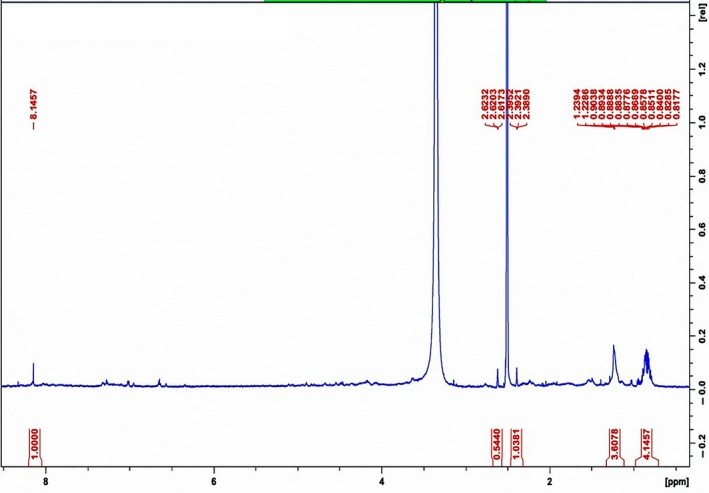
NMR spectrum of BS10.5

The proton analysis of the compound showed NH and OH proton at >8.00 ppm, some –CH_3_ and CH_2_ signals at <2.00 ppm and CH_2_–COO>2.00 ppm but <4.00 ppm (Figure 4). These are suggestive of a peptide backbone of secondary amide, aliphatic chains, and ester linkages, respectively. Chakraborty et al., (2014), gave a similar report from the proton spectra of the lipopeptide extract of *B. vallismortis* JB201 and *B. subtilis* SJ301. The signals of the carbon analysis were inconclusive despite several runs (data not shown).

#### 3.9.3 ESI‐Q‐TOF MS characterization

**Figure 5:**
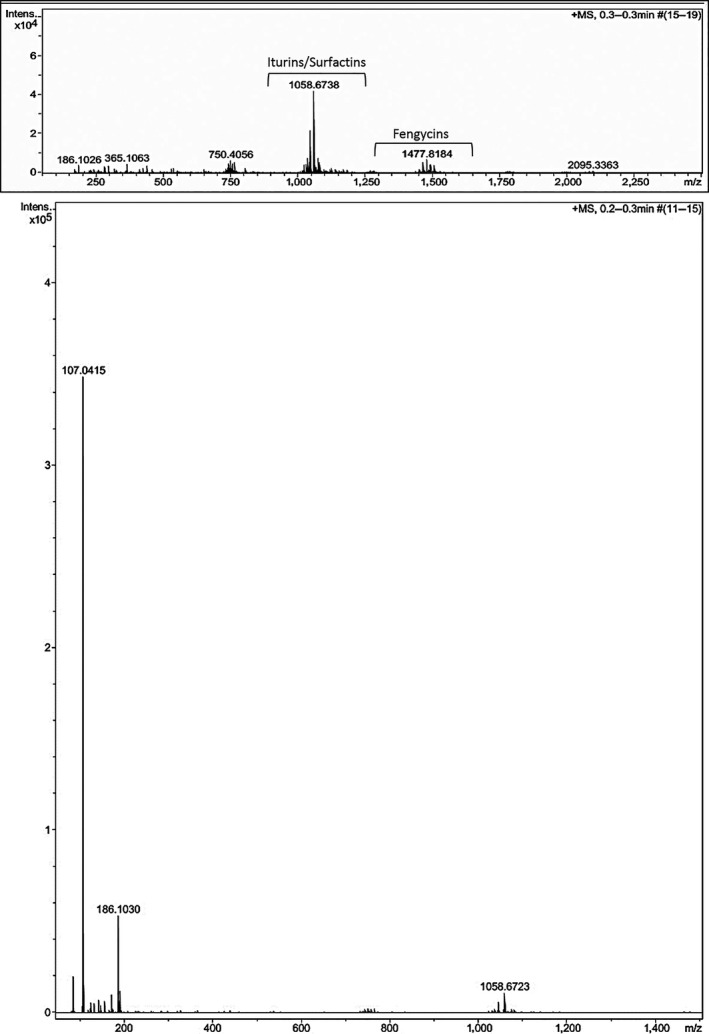
Positive ESI‐Q‐TOF MS spectrum of lipopeptides extract of BS10.5 strain. Clusters of iturin, surfactin, and bacillomycin (*m/z* 1,058.6738/1,058.6723, 1,058.6740), fengycin (*m/z* 1,477.8184), and an unidentified (*m/z* 2,095.3363) molecular ion species are labeled

The ESI‐Q‐TOF MS characterization showed that the BS10.5 lipopeptide extracts contained antifungal iturin, surfactin, and fengycin families (Figure 5). Isolate BS10.5 had six mass ranges starting from *m/z* 186.1026, 365.1063, 750.4056, 1,058.6738, 1,477.8184, and 2,095.3363. Mass 1,058.6738 suggests the possible presence of iturin, surfactin, and bacillomycin fragments, while 1,477.8184 represents the mass fragment of fengycin, respectively (Ben Ayed et al., 2014; Jasim et al., 2016; Nam et al., 2015). This conclusion is based on multiple comparison of literature and fragment matches.

### 3.10 Genome sequence data

**Figure 6:**
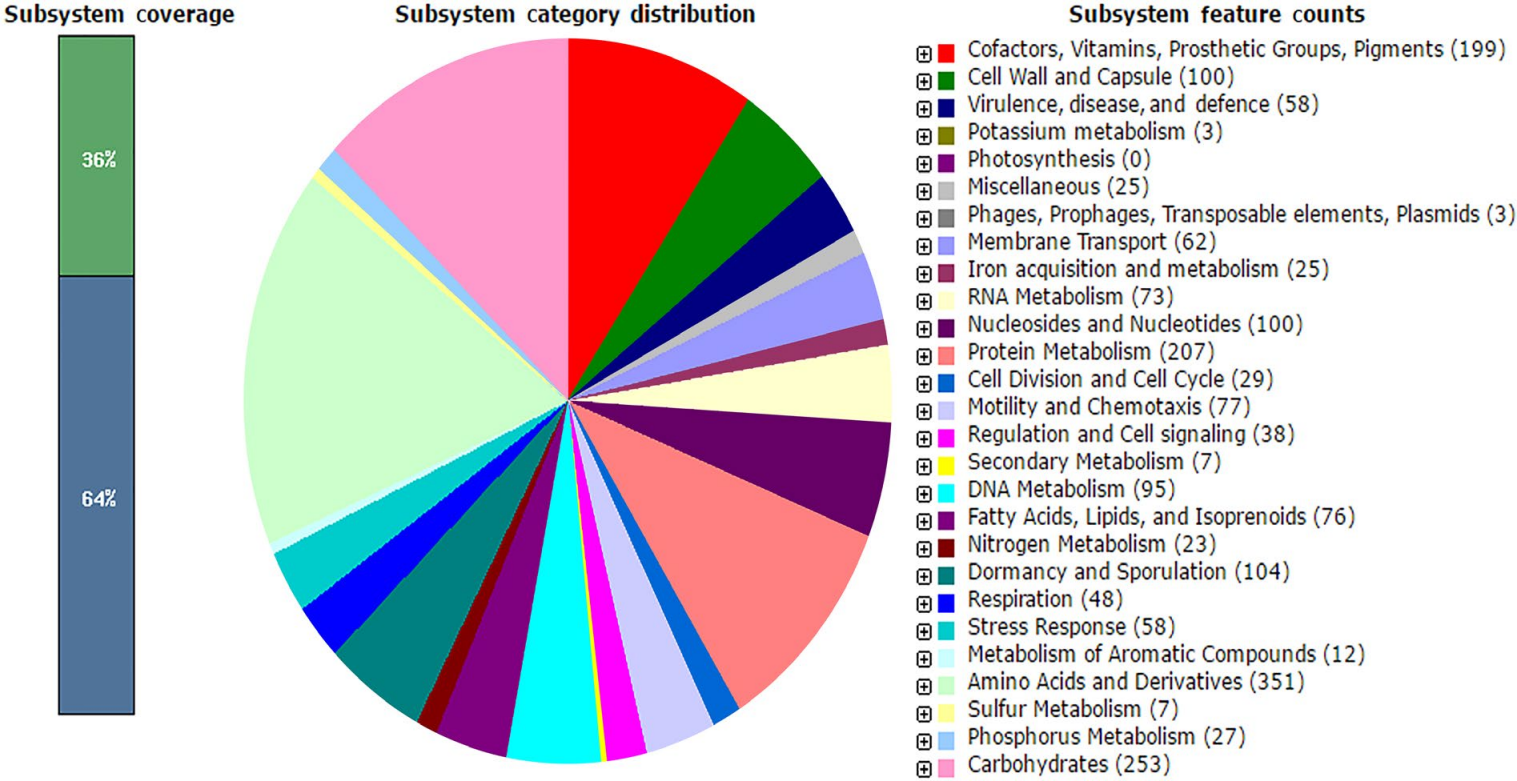
Subsystem summary of the genome *Bacillus velezensis* NWUMFkBS10.5 predicted by SEED Viewer v2.0. Genomic features are colored according to their functional classification types (Overbeek et al., 2014)

Isolate BS10.5 was identified as *B. velezensis* strain NWUMFkBS10.5. The sequencing yielded 7,505,117 clusters and 15,010,234 paired‐end reads with an average read length of 151 bp. After compilation of the data, the genome yielded between 3,905,592 and 3,964,473 bp, and G+C content of 46.4%, showing similarity to known plant growth‐promoting *Bacillus* spp. (Table 5 and Supporting Information Figure S4). Subsystem information of the isolate was predicted by SEED viewer (Overbeek et al., 2014) (Figure 6). Overview of the secondary metabolite biosynthesis gene clusters with antiSMASH v4.0.0rc1 (Weber et al., 2015) revealed that BS10.5 had 16 gene clusters involved in secondary metabolic activities. The clusters were devoted to the synthesis of antimicrobial peptides such as surfactin, mersacidin, fengycin, and oocydin (Table 6). BS10.5 was labeled non‐pathogenic by Pathogenfinder v1.1 web server (Cosentino, Voldby Larsen, Møller Aarestrup, & Lund, 2013).

**Table 5:** Comparison of the *Bacillus velezensis* NWUMFkBS10.5 genome with other *B*. *velezensis* strains

Attributes	*B*. *velezensis* NWUMFkBS10.5	*B*. *velezensis* LM2303	*B. velezensis 157*	*B*. *velezensis* LABIM40	*B*. *velezensis* FZB42	*B*. *velezensis* AS43.3	*B*. *velezensis* UCMB5113	*B*. *velezensis* G341	*B*. *velezensis* LS69	*B*.* velezensis* 9912D
Size of genome (bp)	3,964,473	3,989,393	4,013,317	3,972,310	3,918,589	3,961,291	3,889,532	4,009,746	3,917,761	4,206,167
G+C numbers (%)	46.39	46.68	40.32	46.5%	46.49	46.60	46.71	46.49	46.40	46.03
Number of coding sequences	3,875	3,531	3,369	3,777	3,693	3,861	3,656	3,953	3,643	4,436
Total genes	3,916	3,866	3,789	n.d	3,421	4,037	n.d	4,114	n.d	n.d
tRNA	89	86	87	75	89	89	89	95	72	86
rRNA	13	27	27	7	9	29	10	30	7	27
Number of RNAs	93	n.d	n.d	n.d	117	118	182	161	n.d	n.d

Note

n.d: not documented

**Table 6:** Description and location of BGC in NWUMFkBS10.5 identified in silico

Cluster Identity (ID)	Type	Position/Region	Numbers of BGC	Most Similar BGC predicted	Percentage similarity
Cluster 1 (218.374_ID_10360)	Terpene	42,380–64,263	1	Undefined	—
Cluster 2 (237.089_ID_10346)	Otherks	65,373–106,617	1	Butirosin	7% of genes similar
Cluster 3 (237.089_ID_10346)	Terpene	189,395–210,135	1	Undefined	—
Cluster 4 (237.089_ID_10346)	Transatpks	493,558–579,439	5	Macrolactin	100% of genes similar
Cluster 5 (237.089_ID_10346)	Transatpks—Nrps	808,101–910,775	10	Bacillaene	100% of genes similar
Cluster 6 (237.089_ID_10346)	Transatpks—Nrps	973,335 –1,061,819	10	Fengycin	86% of genes similar
Cluster 7 (228.907_ID_10356)	Transatpks	1–45,825	10	Difficidin	53% of genes similar
Cluster 8 (228.907_ID_10356)	T3pks	161,376–202,527	1	Undefined	—
Cluster 9 (242.696_ID_1037)	Nrps	1–10,331	1	Undefined	—
Cluster 10 (242.696_ID_1037)	Nrps	1–14,180	2	Fengycin	13% of genes similar
Cluster 11 (232.565_ID_10370)	Transatpks	1–23,720	7	Difficidin	26% of genes similar
Cluster 12 (246.163_ID_10350)	Nrps	516,770–569,433	1	undefined	—
Cluster13 (263.57_ID_10354)	Nrps	1–25,748	3	Surfactin	47% of genes similar
Cluster 14 (265.136_ID_10348)	Bacteriocin—Nrps	1,981–68,772	10	Bacillibactin	100% of genes similar
Cluster 15 (265.136_ID_10348)	Other	574,581–615,999	6	Bacilysin	100% of genes similar
Cluster 16 (265.136_ID_10348)	Lantipeptide	766,502–789,690	1	Mersacidin	90% of genes similar

### 3.11 Insights from exploration and in silico mining of *B. velezensis* NWUMFkBS10.5 genome

The in silico analyses revealed that the NWUMFkBS10.5 genome has 16 clusters which harbored over 76 homologous biosynthetic gene clusters (BGC). The genome has four nonribosomal peptide synthetases (NRPSs), two terpenes, three *trans*‐AT PKS (transatpks), a single lantipeptide, a type 3 polyketide synthetase (T3pks), two *trans‐*AT PKS nonribosomal peptide synthetases, a single bacteriocin nonribosomal peptide synthetase cluster and two unidentified ketide synthetases (Table 6 and Supporting Information Figure S5a–k). In Supporting Information Figure S5a–k, the colors symbolize different functional gene types: blue (transport‐related genes), green (biosynthetic genes), red (regulatory genes), and gray (additional genes). In the antismash data, the highest number of biosynthetic genes was found in clusters 5, 6, 7, and 14 (10 each), whereas the PRISM result predicted 18 clusters in the NWUMFkBS10.5 genome. In Table 7, the functions of the predicted biosynthetic compounds are given.

**Table 7:** Overview of the biosynthetic compounds predicted in the NWUMFkBS10.5 genome

Biosynthetic compound	Cluster located	Biomedical/Biocontrol function	Reference
Mycosubtilin	6	Antifungal, hemolytic, and limited antibacterial activity	Duitman et al. (2007), Leclère et al. (2005)
Fengycin and Plipastatin	6	Broad‐spectrum antifungal and antitumoral agent	Cochrane and Vederas (2016), Vanittanakom, Loeffler, Koch, and Jung (1986)
Surfactin	13	Antibacterial, antifungal, antiviral, antimycoplasma, antitumoral, insecticidal, anticoagulant activities, and enzyme inhibitors	Mnif and Ghribi (2015), Ongena and Jacques (2008)
Iturin	6	Antibacterial and antifungal activity	Dunlap et al. (2013), Ongena and Jacques (2008)
Polymyxin	6	Antibacterial, antifungal, and Immuno‐modulating activity	Cochrane et al. (2016)
Sessilin	10	Antifungal activity	D’Aes et al. (2011), Olorunleke, Hua, Kieu, Ma, and Höfte (2015)
Bananamides	10	Unspecified	Nguyen et al. (2016)
Cichopeptin	10	Limited information	Huang et al. (2015)
Viscosin	10	Biosurfactant	Alsohim et al. (2014), Bonnichsen et al. (2015)
Taiwachelin, tolaasinand, orfamide	10	Iron chelation, therapeutic peptide, insecticidal biosurfactant, and elicitor of induced systemic resistance	Andolfi, Cimmino, Cantore, Iacobellis, and Evidente (2008), Jang et al. (2013), Kreutzer, Kage, and Nett (2012), Ma, Ongena, and Höfte (2017)
Mersacidin	16	Antibacterial	Abriouel, Franz, Ben Omar, and Gálvez (2011)
Bacitracin and bacilysin	15	Limited use as animal growth promoter, topical antibiotic, antibacterial, and antifungal	Mousa and Raizada (2015), Phillips (1999)
Bacillibactin, paenibactin, griseobactin, heterobactin, mirubactin, myxochelin, vanchrobactin	14	Iron chelation and anticancer agent	Balado, Osorio, and Lemos (2008), Giessen et al. (2012), Patzer and Braun (2010), Sandy et al. (2010), Wen et al. (2011)
Amylocyclicin	1	Antibacterial and plant growth promoter	Scholz et al. (2014)
Lichenysin	13	Biosurfactant	Grangemard, Bonmatin, Bernillon, Das, and Peypoux (1999)
Basiliskamides	13	Antifungal	Theodore et al. (2014)
Difficidin	11, 7, 4	Broad‐spectrum antibacterial compound	Chen et al. (2009), Mousa and Raizada (2015)
Kalimantacin/batumin and oocydin A	11, 7, 5	Antibacterial and antifungal haterumalide	Matilla, Leeper, and Salmond (2015), Mattheus et al. (2010), Tokunaga et al., (1996)
Sorangicin	11, 7	Antibacterial macrolide antibiotic	Campbell et al. (2005)
Elansolid	7	Antibiotic (Bactericidal)	Steinmetz et al. (2011)
Myxovirescin	7, 5	Antibiotic (Bactericidal)	Xiao, Gerth, Müller, and Wall (2012)
Phormidolide, thiomarinol, and mupirocin	5	Antitumor agent and antibacterial metabolite/clinical antibiotic	Mousa and Raizada (2015), Murphy et al. (2014)
Paenilarvins, tridecaptin, and paenibacterin	6	Antifungal, antitumor agent, and antibiotic (Bactericidal)	Cochrane et al. (2016), Hertlein et al. (2016), Huang, Guo, and Yousef (2014), Huang and Yousef (2014)

### 3.12 Whole genome nucleotide/NCBI biosynthetic gene blast and Pangenome comparison

**Figure 7:**
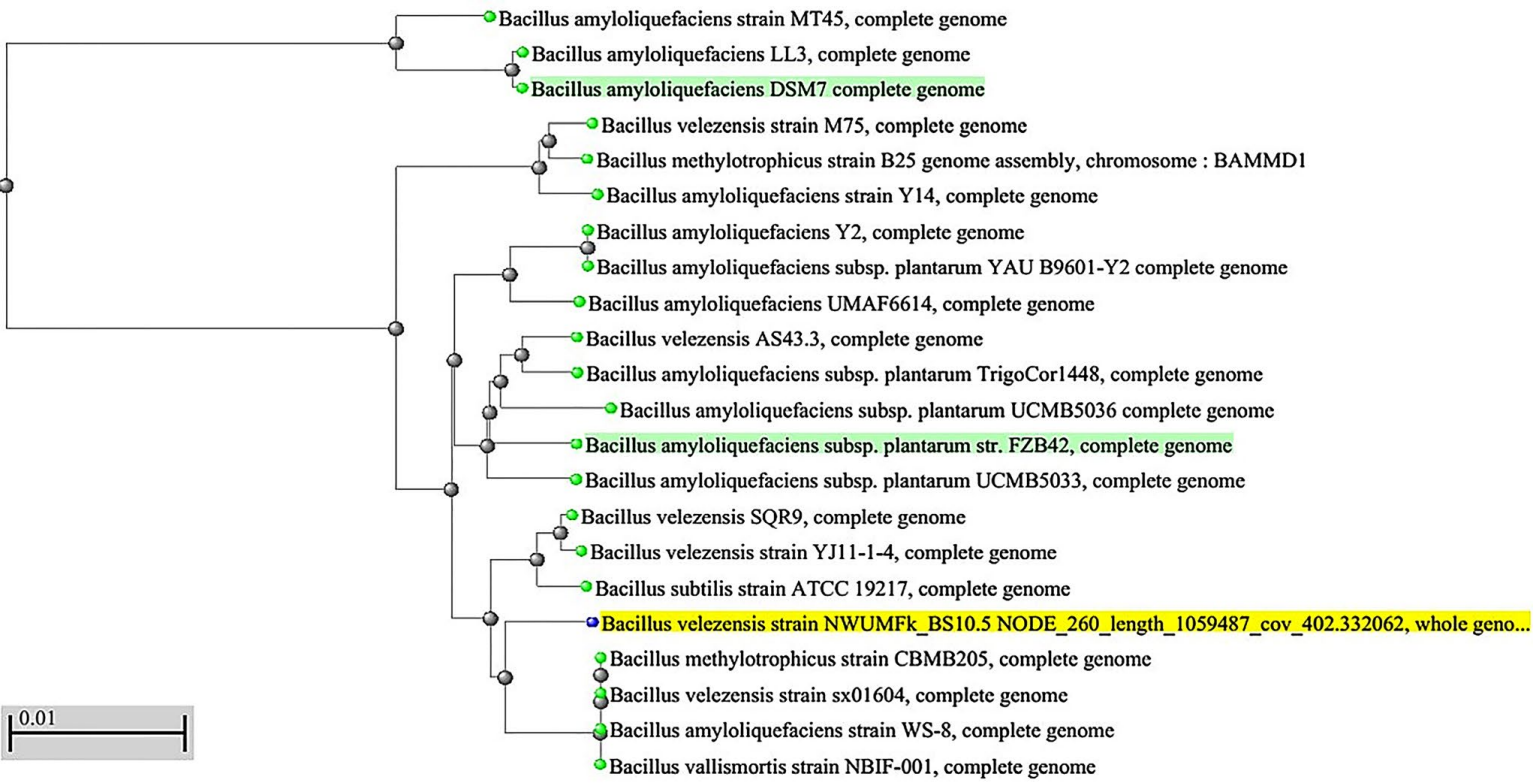
Neighbor‐joining phylogenetic tree from pangenomic sequence of closely related *Bacillus velezensis* strains. Bar, 0.01 substitutions per nucleotide position. Reference strains highlighted green. Strain *B. velezensis* NWUMFkBS10.5 is highlighted yellow

**Figure 8:**
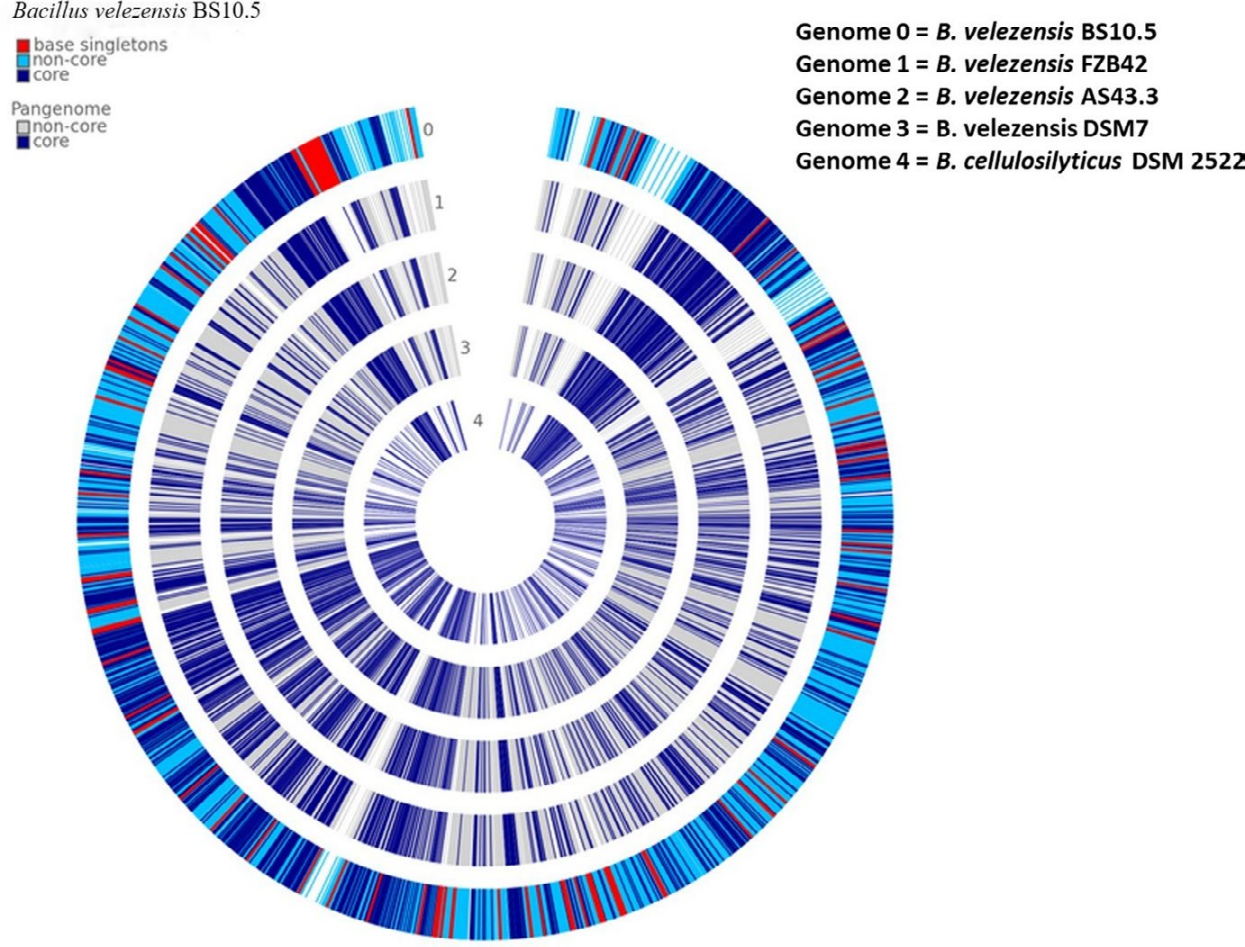
Pangenomic atlas of *Bacillus velezensis* NWUMFkBS10.5, other closely related *B. veleznesis* strains, and an out group B. cellulosilyticus DSM 2522 (genome 4). The similarities and dissimilarities in their core and non‐core genome are indicated as predicted in the left key of the figure. Peculiar genes in strain *B. velezensis* NWUMFkBS10.5 are indicated in its ring as light blue arcs

Firstly, we carried out a genome blast search of NWUMFkBS10.5 to determine its genetic relatedness, and then, we blast searched the genome against selected lipopeptide gene clusters and constructed the phylogenetic trees (Figure 7 and Supporting Information Figure S6). Thirdly, the pangenome comparisons of four established agriculturally and industrially relevant *Bacillus* strains with NWUMFkBS10.5 strains confirmed the distinctive status of BS10.5 and its genetic relatedness (Figure 8).

## 4 DISCUSSION

When in search of beneficial microbial strains for commercialization, key screening approaches and sequential steps have been identified that lead to selecting robust and effective candidates (Köhl, Postma, Nicot, Ruocco, & Blum, 2011). A comprehensive in vitro analysis starting from the culturing stage to molecular identification of action mechanisms and backed up by in silico genome exploration of potential candidates provides strains with better in planta viability and efficacy (Adeniji & Babalola, 2018). In this study, out of the 200 isolates initially screened for antagonism against the fusarium pathogens, 11 isolates showed consistent antagonism and among 11 isolates, isolate BS10.5 exhibited the strongest and most consistent antagonism considering the conditions. Applying biocontrol agents as a bioprotective coating prior to planting of crop seedlings has been demonstrated in previous reports (Yang et al., 2015). The antifungal result of this study demonstrates that our *Bacillus* isolates are candidates for preplanting and post‐harvest bioprotective inoculants. The reduction in fungal mycelia growth (Tables 1 and 2) shows they could protect plants against the onset of fusariosis. Members of the *Bacillus* sp. exhibit remarkable biocontrol activity against phytopathogens due to the production of the lipopeptide group of antibiotics (surfactin, fengycin, bacillomycin, and iturin) (Guo et al., 2014; Vitullo, Pietro, Romano, Lanzotti, & Lima, 2012). We detected these groups in some of our *Bacillus* isolates.

Production of biosurfactants has been reported for many *Bacillus* strains (Chen, Zhang, Fu, Li, & Wang, 2016; Plaza, Chojniak, Rudnicka, Paraszkiewicz, & Bernat, 2015). The four biosurfactant producers (BS1.1, BS3.5, BS8.6, and BS10.5) identified in the study have potential agricultural, industrial, biological, and medical applications (Table 3). Biosurfactants are biologically surface‐active agents with both lipophilic and hydrophilic moieties (Kosaric, Gray, & Cairns, 1987). They act by decreasing the surface and interfacial tension between individual molecules in contact, forming selective ionic pores in membrane bilayers. With biosurfactants having antiviral, antibacterial, hemolytic, antifungal, and anti‐insecticidal properties (Mnif & Ghribi, 2015), the antimicrobial mechanisms of these surfactant producers are further worth exploiting.

Culture‐free extracts from *B. velezensis* antagonists have been used in bioprotection assays through several modes of application such as seed treatments and pour plate mixtures (Cao et al., 2018). These culture‐free extracts are of beneficial importance when produced in sufficient volume during fermentation processes. The supernatant contains diverse metabolites such as indole‐acetic acid (IAA), antibiotics, lytic enzymes, iron chelating compounds, and hormones depending on the microbial growth phase and culture conditions (Ahmad, Ahmad, & Khan, 2008; Chen et al., 2014). The secretion and accumulation of surfactin occur at the logarithmic phase while the synthesis of iturin occurs during the stationary phase of growth (Leclère et al., 2005). The antimicrobial activity shown by the cell‐free supernatants of isolate BS10.5 suggests that the secondary metabolite of the isolate can also be of immense industrial benefit when purified in sufficient quantity.

Having employed a dose‐dependent assay to determine the concentrations at which the pathogens were most sensitive to BS10.5 extracts, we report that the growth of the pathogens was retarded relative to increased concentration of BS10.5 extract. At 30 µl, three of the pathogens (PA, MC, and KP) showed no sensitivity. This gives an indication of the lowest possible concentration at which the pathogens will remain susceptible. Dose‐dependent effects of lipopeptides have been reported elsewhere (Falardeau, Wise, Novitsky, & Avis, 2013). A detailed assay might however be needed to confirm the minimum inhibitory or cidal dose of the extract. Commercial fungicides containing nystatins, triazoles, and amphotericins have been used as controls to compare sensitivity of *Fusarium* to fungal antibiotics (Arutchelvi & Doble, 2010; Girija, Duraipandiyan, Kuppusamy, Gajendran, & Rajagopal, 2014), and *Fusarium* spp. are less susceptible to amphotericin than to nystatins and triazoles (Deepak & Jayapradha, 2015). Our lipopeptide extract showed excellent inhibitory activity in comparison with the fungicides we utilized in this study. Our results correlate with other reports showing comparative multi‐antipathogenic effects of crude, partially purified, or purified extracts of bacterial antagonists (Chen et al., 2010; Mousa et al., 2015). The action mechanisms of microbial lipopeptides reduce the possibility of microbes developing resistance to them, while conventional antibiotics are prone to resistance.

The FTIR, NMR, and ESI‐Q‐TOF MS results provide further evidence that the lipopeptides (surfactin, iturin, and fengycin) prominently reported in *Bacillus* spp. are responsible for the biocontrol activity of NWUMFkBS10.5. Reports showing non‐synergistic and synergistic activity of lipopeptides against *Fusarium* members are available (Koumoutsi et al., 2004; Xu et al., 2013), and *Bacillus* spp. with conserved genes for producing multiple lipopeptides are not rare (Jemil et al., 2017). BS10.5 harbored multiple lipopeptide antibiotic genes. The ESI‐MS was effective in detecting the presence of these compounds, but we could not speculate what compound produced the signal at *m/z* 2,095.3363. Furthermore, except for ericin S with 3,351.543 *m/z* (Palazzini et al., 2016), high signals of this range have been rarely reported for *Bacillus* lipopeptides. Fragment spectra of surfactin and its homologs (C13, 14, and 15) are mostly detected at 1,030.8, 1,044.8, 1,046.8, 1,058.8, 1,060.8, and 1,074.8, that of bacillomycin (C14, 15, 16, and 17) at 1,031.7, 1,045.7, 1,053.7, 1,059.7, 1,067.7, 1,069.7, 1,081.7, 1,083.7, 1,097.7, 1,095.7, 1,111.7, 1,527.8, 1,529.9, and 1,543.8, those of fengycins (C15, 16, and 17) at 1,449.9, 1,463.9, 1,471.9, 1,477.9, 1,485.9, 1,487.9, 1,491.8, 1,499.9, 1,501.9, 1,505.8, 1,513.9, and 1,515.9 (Koumoutsi et al., 2004). The majority of these lipopeptides have a large variety of isoforms which sometimes present challenges in distinguishing them (Chen et al., 2014). Our aim in this study was not to resolve each lipopeptide type into its specific isoforms, but to detect the different groups of lipopeptides present in the metabolite and confirm that the gene clusters detected are metabolically functional.

The presence of surfactin, bacillomycin, iturin D, and fengycin D biosynthetic genes, detected in the BS10.5 amplicons during PCR analysis, was confirmed from the WGS data. The BS10.5 genome analysis showed similarity identity above 85% for some biosynthetic genes clusters (BGC). Technically, BGC or biosynthetic genes are considered to be present when the similarity index is 65% and above (Van Der Voort et al., 2015). However, our report here also shows that a low percentage similarity of a BGC does not signify the absence of the predicted BGC in the genome being analyzed and this correlates with our identification of iturin gene during PCR, despite its predicted similarity identity in cluster 6 (BGC0001098) being below 65%. Coding region specific for fengycin is predicted to be present in cluster 6 at 86%, bacillomycin at 66%, and iturin at 53% similarity (Table 6). These genes were also detected by specific primers during the PCR‐gel electrophoresis and ESI‐Q‐TOF MS analysis.

The *B. velezensis* sp. is categorized as heterotypic synonyms of *B. amyloliquefaciens* subsp. *plantarum* FZB42T, *B. methylotrophicus* KACC 13015T, and *B. oryzicola* KACC 18228 based on DNA‐DNA hybridization values >84% (Dunlap, Kim, Kwon, & Rooney, 2016; Fan, Blom, Klenk, & Borriss, 2017). The in silico result showed that our *B. velezensis* NWUMFkBS10.5 strain closely shares some phenotypic and genotypic traits with several of the commercially established plant growth‐promoting strains within the *Bacillus* genus (Table 6, Supporting Information Figures S4 and S7a,b). We avoided relying only on the structural and functional metabolic capacity of well‐studied or representative *B. velezensis* strains to deduce the metabolic capacity of NWUMFkBS10.5. The approach has been reported to have limitations, because experimental data have shown that in some species, new genes are discovered after comparing the genomes of newly sequenced strains and reference strains (Medini et al., 2005).

We applied the pangenomic analysis to locate genes that were responsible for metabolic activity and genes that were dispensable to the survival of NWUMFkBS10.5. The analysis showed that the strain shared core resemblance with the other analyzed *Bacillus* isolates. Additionally, NWUMFkBS10.5 genome had five undefined BGC clusters (1, 3, 8, 9, and 12) (Table 6) and did not contain amylolysin unlike other *B. velezensis* strains (Liu et al., 2017; Pan et al., 2017). In Table 5 and Figure 7a, our *B. velezensis* NWUMFkBS10.5 genome did not show close phylogenomic clustering with the popular industrially and agriculturally applied *B. velezensis* strains (*B. velezensis* FZB42, AS43.3, UCMB5033, DSM7, Trigorcor 1448, and M75). The iturin cluster was predictably found in cluster 6, subcluster BGC0001098 (Supporting Information Figure S5h) of NWUMFkBS10.5, even though the NWUMFkBS10.5 blast did not branch directly with any of the iturin operons (Supporting Information Figure S6). It, however, contained putative gene clusters encoding sporulation, biofilm formation, and antibiotic synthesis, (macrolactin, bacilysin, bacillibactin, surfactin, bacillopeptin, bacillaene, iturin, fengycin, and difficidin) that could also be found in these strains (Pandin et al., 2018) (Tables 6 and 7). Gene clusters for amylocyclicin (detected in *B. velezensis* FZB42 and *B. velezensis* LS69) (Liu et al., 2017; Scholz et al., 2014), butirosin (detected in *B. velezensis* LM2303) (Chen, 2017), ericin (detected in *B. velezensis* RC 218) (Palazzini et al., 2016), and lignin degradation (Chen et al., 2018) were located in NWUMFkBS10.5 genome.

Remarkably, biosynthetic clusters predicted to synthesize compounds such as sessilin, bananamides, cichopeptin, taiwachelin, tolaasin, basiliskamides, kalimantacin, and mersacidin were however identified in strain NWUMFkBS10.5 (Table 6). These compounds are rarely documented for *B. velezensis* strains, and this suggests possible evolutionary pressure for the development of competitive antimicrobials among the *B. velezensis* group. Our goal in this study was to provide a bioprotective *Bacillus* strain for maize against the onset of fusariosis. *B. velezensis* NWUMFkBS10.5 was prudentially selected because it harbored functional and metabolically active gene clusters responsible for synthesizing diverse beneficial compounds. To date, no standard effective chemical fungicide exists for the treatment of fusariosis in South Africa. To the best of our knowledge, this is a rare report describing the genomic and biocontrol potential of a native *B. velezensis* strain peculiar to Africa. Although we did not corroborate our results with in planta experiments in this study, the exhaustive nature of this combinatorial in vitro study affirms the in planta viability of *B. velezensis* NWUMFkBS10.5. Lastly, the information gathered on NWUMFkBS10.5 will be valuable for its biotechnological manipulation and its probable development into a biofungicide in South Africa.

## CONFLICT OF INTEREST

The authors declare that they have no conflict of interests.

## AUTHORS CONTRIBUTION

AAA designed and performed the in vitro assays/experiments, molecular analysis, in silico genome analysis, analyzed the data, and drafted the manuscript. OSA contributed to the chemical characterization and its data interpretation. OOB participated in the experimental design, supervised the work, and provided funding. All authors contributed to the drafts, final version of the paper, and approved submission.

## ETHICS STATEMENT

This article does not contain any studies with human participants or animals performed by any of the authors.

## Data Availability

The accession numbers of the 16S rDNA nucleotide sequences have been deposited at the NCBI GenBank (Supporting Information Table S4). Whole Genome Shotgun project is deposited at DDBJ/ENA/GenBank under the accession NZ_NITU01000037.1. The version described in this chapter is version NZ_NITU01000037.1 (GCF_002204665.1). The BioProject and BioSample designation for this project are PRJNA388288 and SAMN07174738. The datasets generated during and/or analyzed during the current study are available on request.
