# Supplementary material for: Selecting lipopeptide‐producing, Fusarium‐suppressing Bacillus spp.: Metabolomic and genomic probing of Bacillus velezensis NWUMFkBS10.5
Source: Microbiologyopen. 2018 Oct 25;8(6):e00742. doi: 10.1002/mbo3.742 (PMC6562122; doi:10.1002/mbo3.742)
Supplement: Supplementary file 1 [file MBO3-8-e00742-s001.docx]

**ORIGINAL ARTICLE:**

**Title:** Selecting Lipopeptide Producing, *Fusarium* Suppressing *Bacillus* spp.: Metabolomic and Genomic Probing of *Bacillus velezensis* NWUMFkBS10.5

Adetomiwa A. ADENIJI ^1, 2^, Oluwole S. AREMU ^3^ and Olubukola O. BABALOLA ^1, 2*^ (orcid: 0000-0003-3417-2700 ^1, 2^, 0000-0002-1542-8267 ^3^ and 0000-0003-4344-1909 ^1, 2*^)

Department of Biological Sciences^1, 2^, Food Security and Safety Niche Area^1,2^, Department of Chemistry^3^, Faculty of Natural and Agriculture Science, North-West University, Mmabatho, South Africa

^*^Correspondence: olubukola.babalola@nwu.ac.za; Tel: +27183892568, Fax: +27183892134

**SUPPLEMENTARY FIGURES**

**Table S1:** Colonial characteristics of rhizobacteria antagonist strains in HiChrome *Bacillus* agar

| Organisms (ATCC) | Growth | Color of Colony |
| --- | --- | --- |
| *B. subtilis* (6633) | +/++ | light green to green colonies |
| *B. cereus* (10876 | +++ | light blue, large, flat colonies with blue center |
| *B. thuringiensis* (10792) | +++ | light blue, large, flat colonies with irregular margins |
| *B. megaterium* (14581) | +++ | yellow, mucoid colonies |
| *B. coagulans* (7050) | +++ | pink, small, raised colonies |

**Table S2:** Geographic sites and numbers of *Bacillus* isolates selected from samples collected

| Sample location coordinates | *Bacillus* isolate codes | Total no. distinct colonies selected | No. isolates showing any antagonism | Best isolates with antagonistic potential |
| --- | --- | --- | --- | --- |
| Morwatshetlha (W) (25.8842° S, 25.5089° E) | MOREBS1.1,2,3,5,… 20 | 20 | 2 | *MORWBS1.1 |
| Klipirani (25°52'60" S and 27°25'60" E) | KLIBS2.1,2,3,5,... 20 | 20 | 2 | *MARBS4.6 |
| Harbeestlaagte (A) (26°11'44.2"S 25°24'25.0"E) | HARABS3.1,2,3,5,… 20 | 20 | 2 |  |
| Maretsane (26°08'58.7"S 25°25'27.3"E) | MARBS4.1,2,3,5,… 20 | 20 | 3 |  |
| Vergelee bray (A)( 25°46'39.5"S 24°11'26.3"E) | VERABS5.1,2,3,5,… 20 | 20 | 1 | *VERBS5.5 |
| Morwatshetlha (E) (25.8842° S, 25.5089° E) | MORWBS6.1,2,3,5,… 20 | 20 | 4 | *MOREBS6.3 |
| Vergelee bray (B) ((25°46'32.6"S 24°11'22.5"E)) | VERBBS7.1,2,3,5,… 20 | 20 | 2 |  |
| Molewane (25.8000° S, 25.7333° E) | MOLBS8.1,2,3,5,… 20 | 20 | 4 | *MOLBS8.5 and *MOLBS8.6 |
| Harbeestlaagte (B) (26°11'44.2"S 25°24'25.0"E) | HARBBS9.1,2,3,5,… 20 | 20 | 3 |  |
| North West University (25°49'16.8"S 25°36'52.8"E) | NWUMFkBS10.1,2,3,5,… 20 | 20 | 3 | *NWUMFkBS10.5 |

**Table S3:** PCR amplification and target genes

| Primer Sequence (5’-3’) | Gene Name | Biosynthetic substance | Annealing Conditions | Expected Amplicon size (bp) | Reference |
| --- | --- | --- | --- | --- | --- |
| *ItuD1f* ATGAAGATTTACGGAATTTA  *ItuD1r* TTATAAAAGCTCTTCGTACG | *ItuD* | Iturin A | 1 min for 55◦C | 647 | Gond et al., 2015 |
| *BacF* GGGAAACCGGGGCTAATACCGGAT  R1378 CGGTGTGTACAAGGCCCGGGAACG | 16s rDNA | - | 90s for 65◦C | 1300 | Garbeva et al., 2003 |
| *Af2F* GAATAYMTCGGMCGTMTKGA  *Tf1R* GCTTTWADKGAATSBCCGCC | NRPS | Fengycins | 30s for 45◦C | 443, 452,455 | Tapi et al., 2010; Ayed et al., 2014 |
| *As1F* CGCGGMTACCGVATYGAGC  *Ts2R* ATBCCTTTBTWDGAATGTCCGCC | NRSP | Surfactin | 30s for 43◦C | 419, 422, 425, 431 | Tapi et al., 2010; Ayed et al., 2014 |
| *FenD1f* TTTGGCAGCAGGAGAAGTTT  *FenD1r* GCTGTCCGTTCTGCTTTTTC | *FenD* | Fengycin | 1 min for 55◦C | 964 | Gond et al., 2015 |
| *sfp-f* ATGAAGATTTACGGAATTTA  *sfp-r* TTATAAAAGCTCTTCGTACG | *sfp* | Surfactin | 1 min for 55◦C | 675 | Gond et al., 2015 |
| *Sur3f* ACAGTATGGAGGCATGGTC  *Sur3r* TTCCGCCACTTTTTCAGTTT | *SrfC* | Surfactin | 1 min for 55◦C | 441 | Gond et al., 2015 |
| *Bacc1f* GAAGGACACGGAGAGAGTC  *Bacc1r* CGCTGATGACTGTTCATGCT | *Bam C* | Bacillomycin D | 1 min for 55◦C | 875 | Gond et al., 2015 |
| ipdcF GAAGGATCCCTGTTATGCGAACC  ipdcR CTGGGGATCCGACAAGTAATCAGGC | *IAA* | *Indole pyruvate*  *decarboxylase* |  | 1700 | Kim et al., 2013 |
| ACC4a CAGCAGGAAAAGGATTTGGG  ACC4b ACTCCACTGAATTGAACCCG | *acc* | *ACC3*  *deaminase* |  | 850 | Kim et al., 2013 |

**Table S4:** Blast results of the *Bacillus* isolates partial 16S rDNA gene sequence alignment and identity search on the NCBI webpage

| Isolate | Accession no | Blast ID (closest cultured similarity match) | Accession no | Similarity | E- value |
| --- | --- | --- | --- | --- | --- |
| KLIBS2.7 | MF098606 | *Bacillus wiedmannii* | KU198626.1 | 100 | 0.0 |
| VERABS5.5 | MF098608 | *Bacillus cereus* | AE016877.1 | 100 | 0.0 |
| MORWBS6.3 | MF098609 | *Bacillus thuringiensis* | CP020754.1 | 100 | 0.0 |
| MOLBS8.5 | MF098610 | *Bacillus toyonensis* | KJ812450.1 | 100 | 0.0 |
| MOLBS8.6 | MF098611 | *Bacillus thuringiensis* | CP021061.1 | 100 | 0.0 |
| MORWBS1.1 | MF098612 | *Bacillus cereus* | KJ812418.1 | 100 | 0.0 |
| NWUMFkBS10.5 | KX353617.1 | *Bacillus velezensis* | KY694464.1 | 100 | 0.0 |

**Table S5:** Inhibition rates of cell free supernatants of BS10.5 on microbial pathogens

|  | Pathogens | | | | | |
| --- | --- | --- | --- | --- | --- | --- |
| Test substances | Fcul | Fg | KP | PA | EF | BC |
| BS10.5 | **+++** | **++++** | **++** | **++** | **+++** | **++++** |
| Ciprofloxacin | ND | ND | **+** | **+++** | **++** | **++** |
| Tetracycline | ND | ND | **+++** | **-** | **++** | **++** |
| Nystatin | **+++** | **+++** | ND | ND | ND | ND |

_Fcul =_ *_F. culmorum_*_; Fg =_ *_F. graminearum_*_; KP =_ *_Klebsiella pneumonia_* _ATCC 25923, PA =_ *_Pseudomonas aeruginosa_* _ATCC 27853 EF =_ *_Enterococcus faecalis_* _ATCC 29212, BC =_ *_Bacillus cereus_* _ATCC 10876._ **_+ = zone of inhibition_**_,_ **_-_** _= no inhibition zone: (_**_+_** _= weak;_ **_++_** _= moderate;_ **_+++_** _= good;_ **_++++_** _= high, represent relative inhibition rates of supernatants on growth of each pathogen on the Lb-PDA agar to the level of 10-29%, 30–49%, 50–69% and ≥70%, respectively._

**
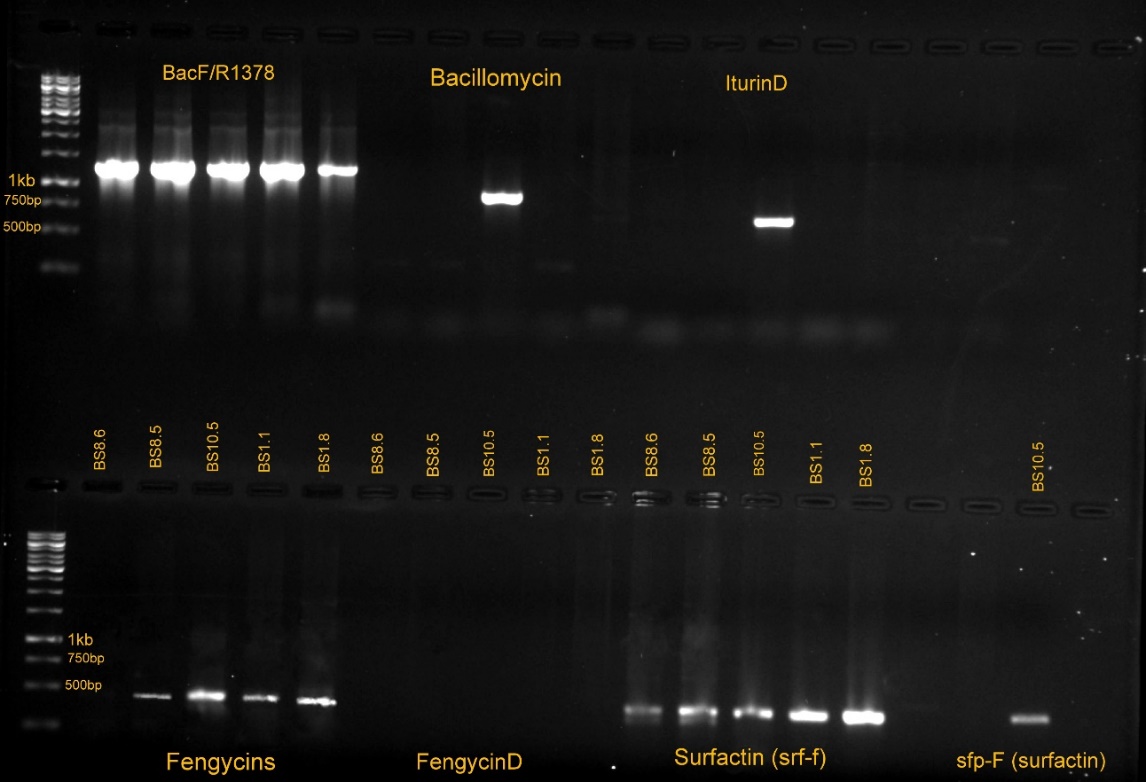
PCR amplification and target genes**

**Fig. S1:** Agarose gel photograph showing amplicons of functional genes in selected *Bacillus* strains with consistent antifungal activity. PCR amplification by degenerate primers: Upper Lane:- *BacF* lane 2-6, *Bacc* lane 7-11, *ItuD* lane 12-16, *sfpF* lane 17-18. Lower lane B:- Fengycins lane 2-6, *FenD* lane 7-11, As1-F lane 12-16, *sfp* F lane 17-19.


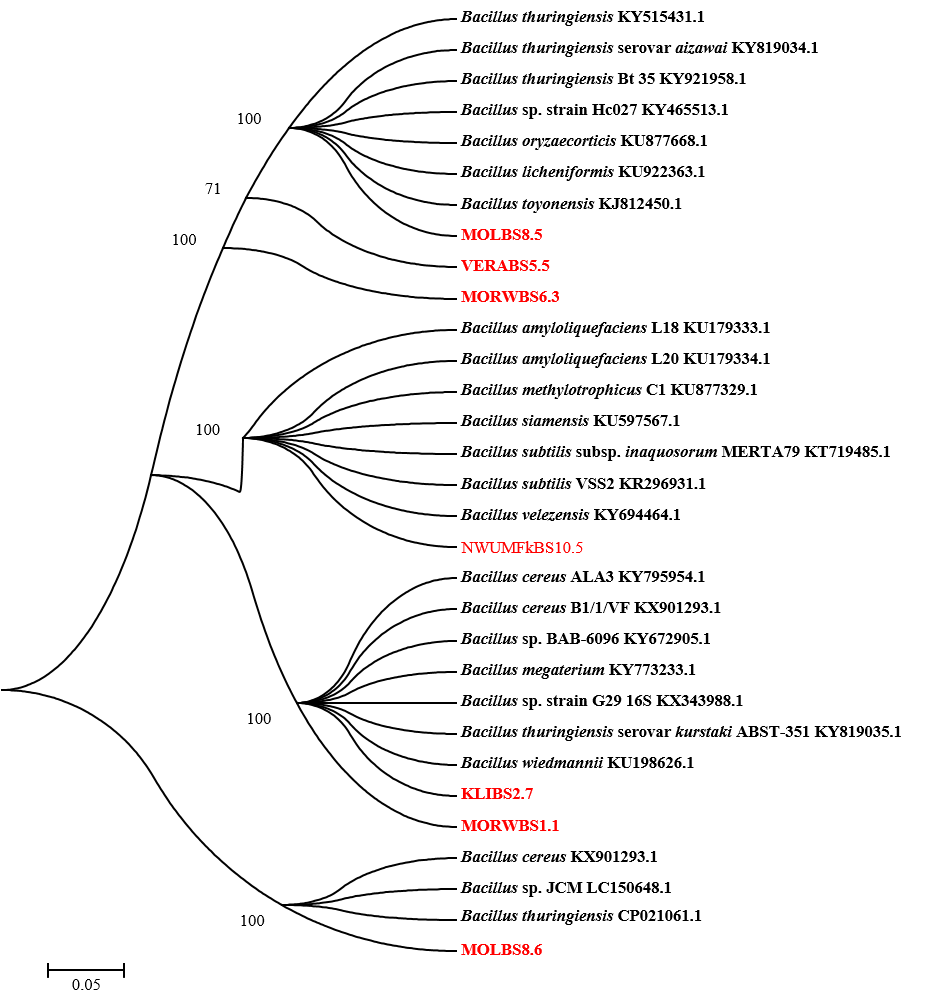


**Fig. S2:** Evolutionary relationships of taxa using Neighbor-Joining method of phylogenetic tree based on partial 16S rDNA gene sequence, showing the phylogenetic relationships between *Fusarium* inhibitors and the most closely related strains from the GenBank. Only values > 50% are shown.


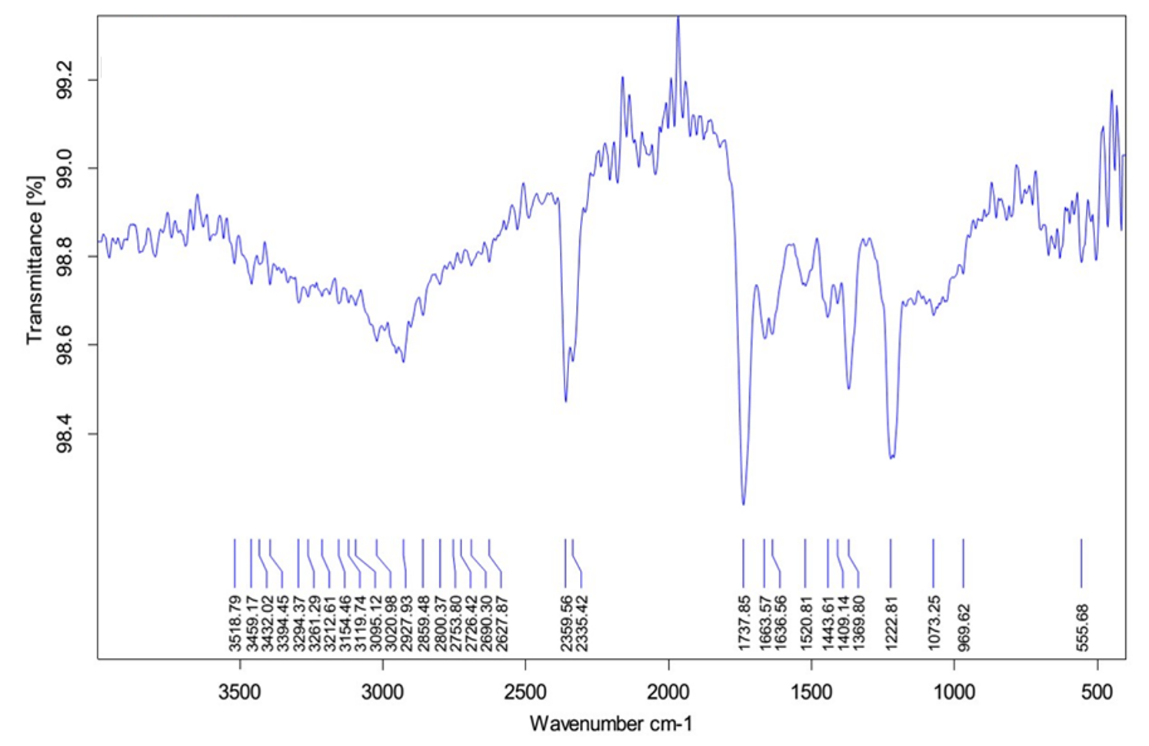


**Fig. S3:** FTIR peaks of lyophilized BS10.5 extract


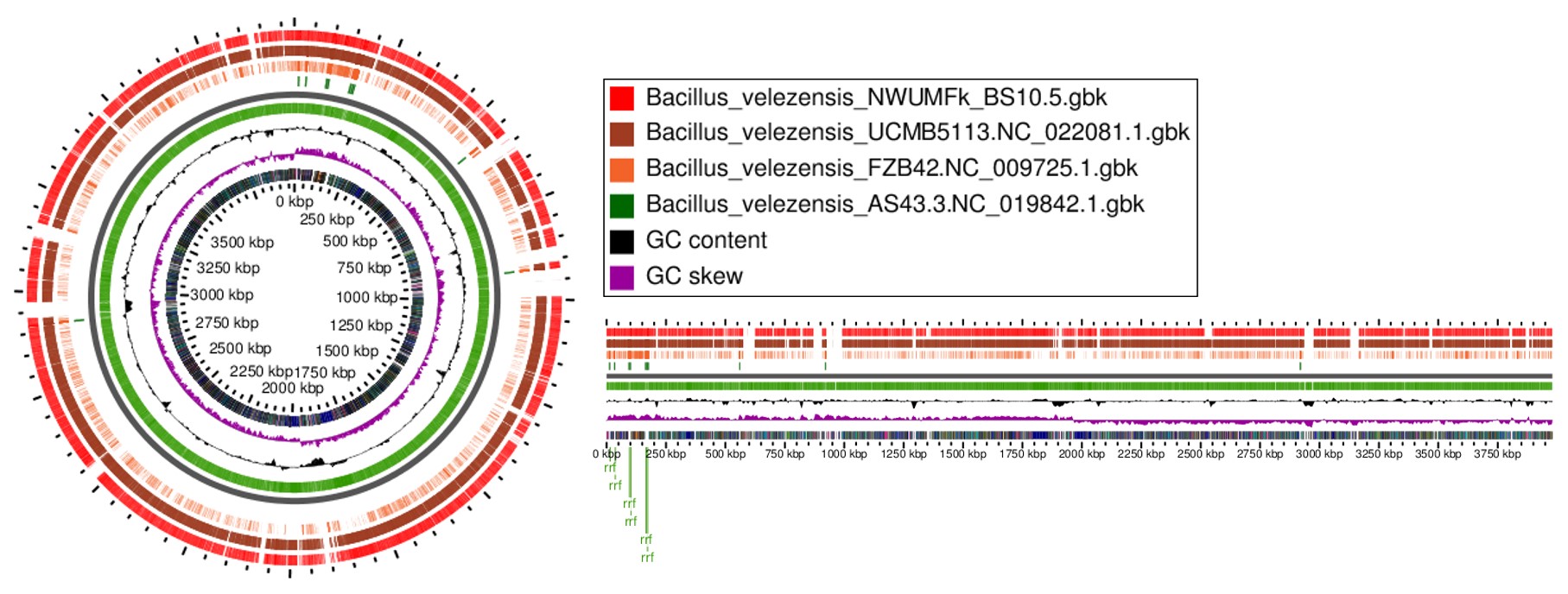


**Fig. S4:** Blast atlas of NWUMFk BS10.5 and closely related *Bacillus velezensis* strains*.* Ribosome recycling factor is shown on the linear map as *rrf.* Genome features compared include rRNA, tRNA, genes and coding sequences (CDS). Circular map was generated by GView Server (GPLv3) (Petkau et al., 2010).

**Molecular annotation of the predicted sixteen clusters identified in the *Bacillus velezensis* NWUMfkBS10.5 genome.**

*
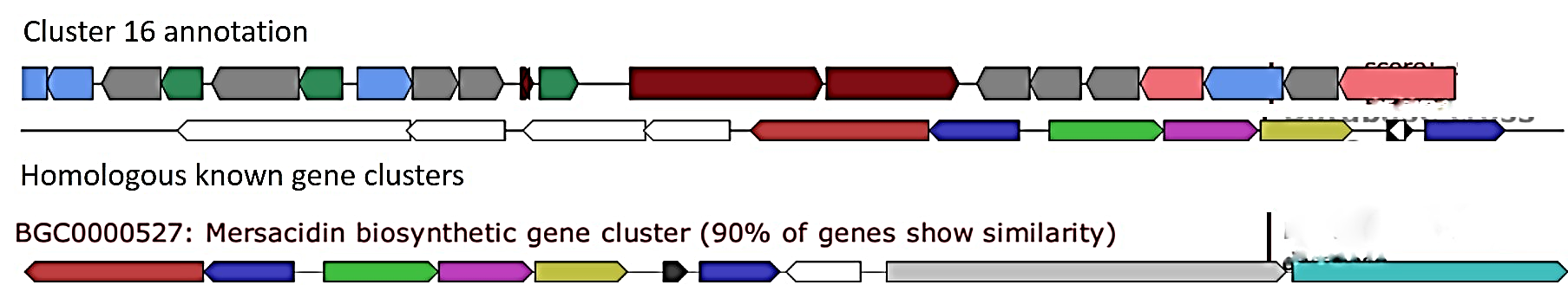
*

**Fig. S5a:** Mersacidin was the major BGC predicted from cluster 16 of the BS10.5 genome


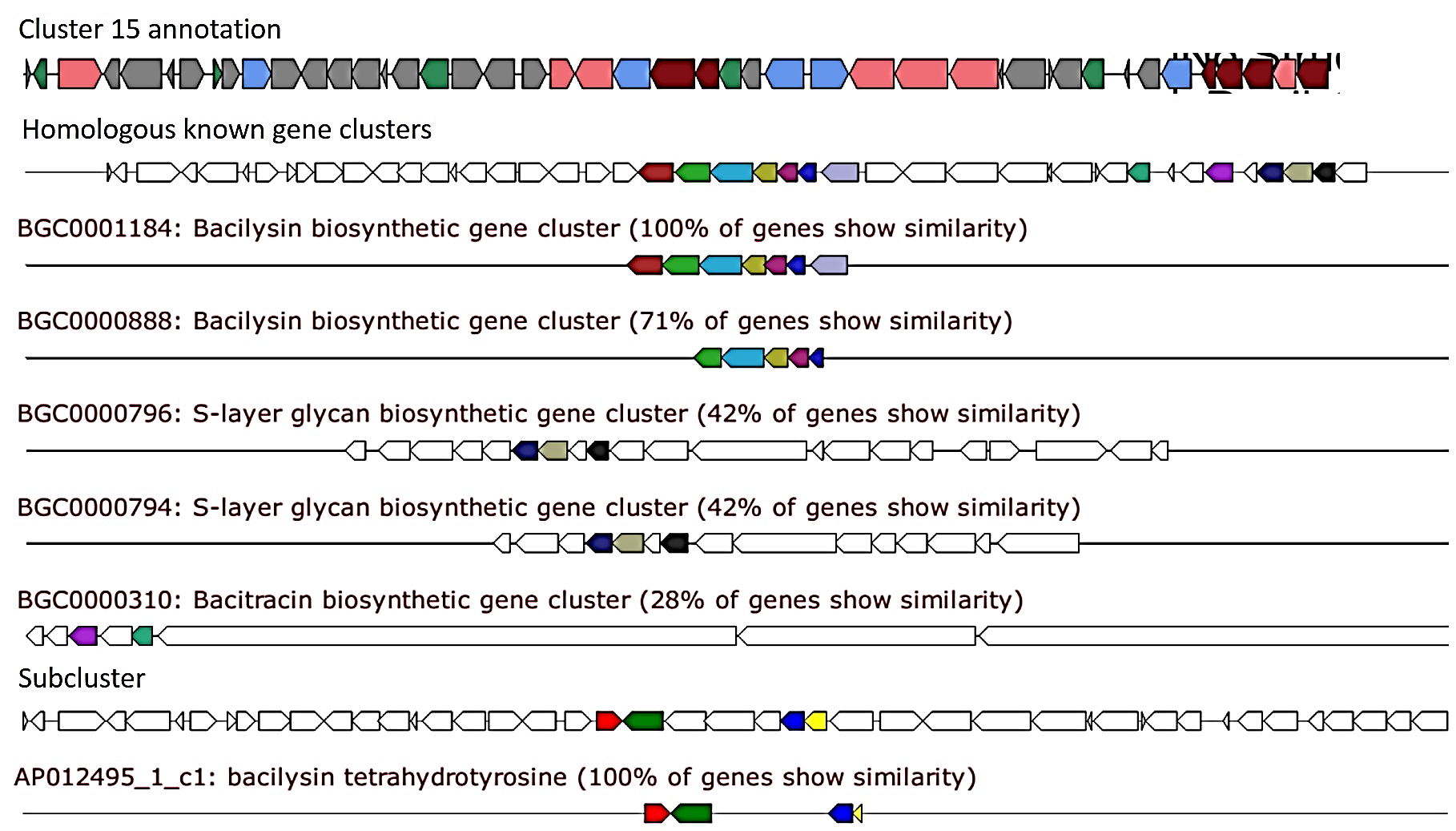


**Fig. S5b:** Bacilysin, S-layer glycan, Bacitracin were the major BGC predicted from cluster 15 of the BS10.5 genome


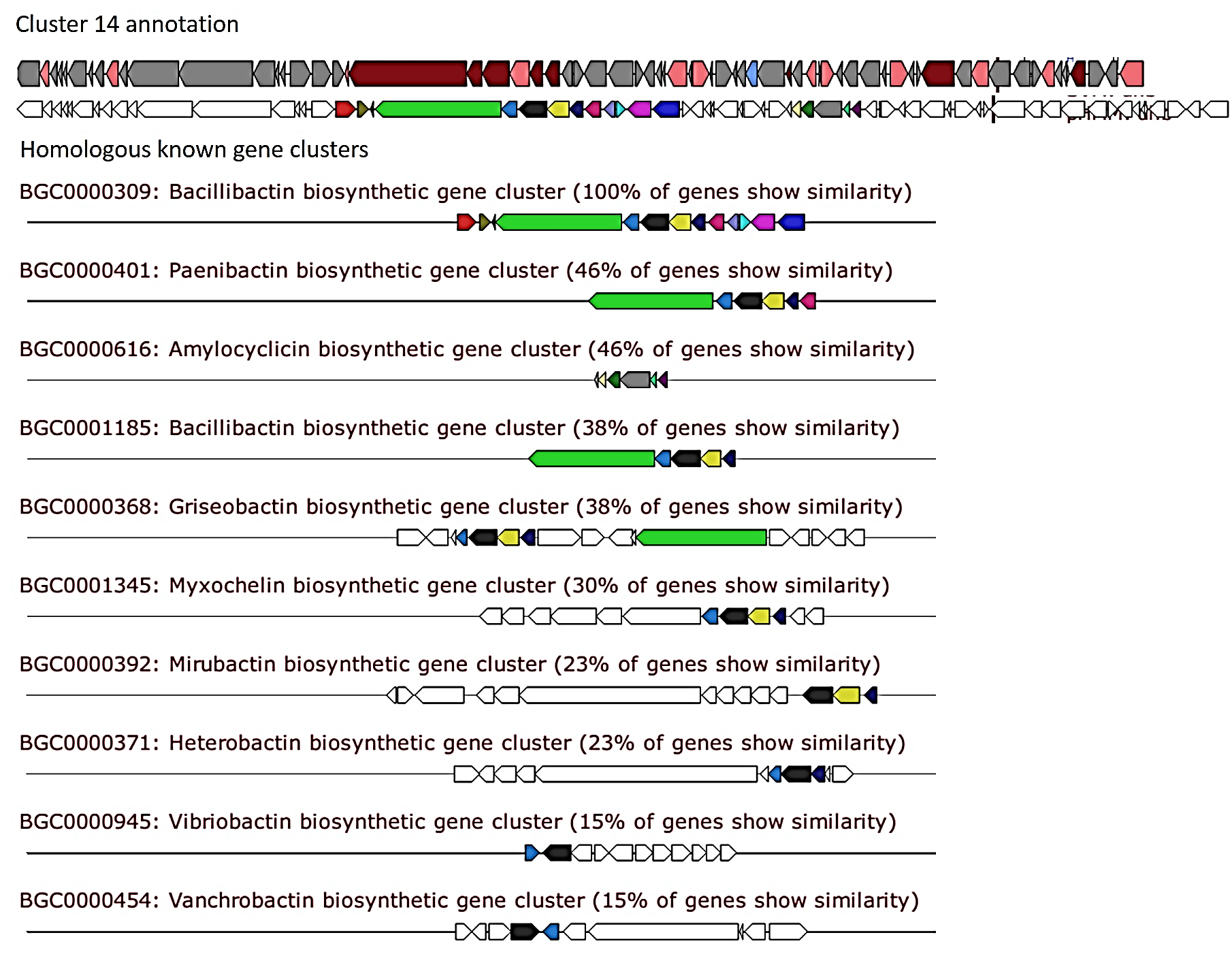


**Fig. S5c:** Major BGC predicted from the cluster 14 of the BS10.5 genome


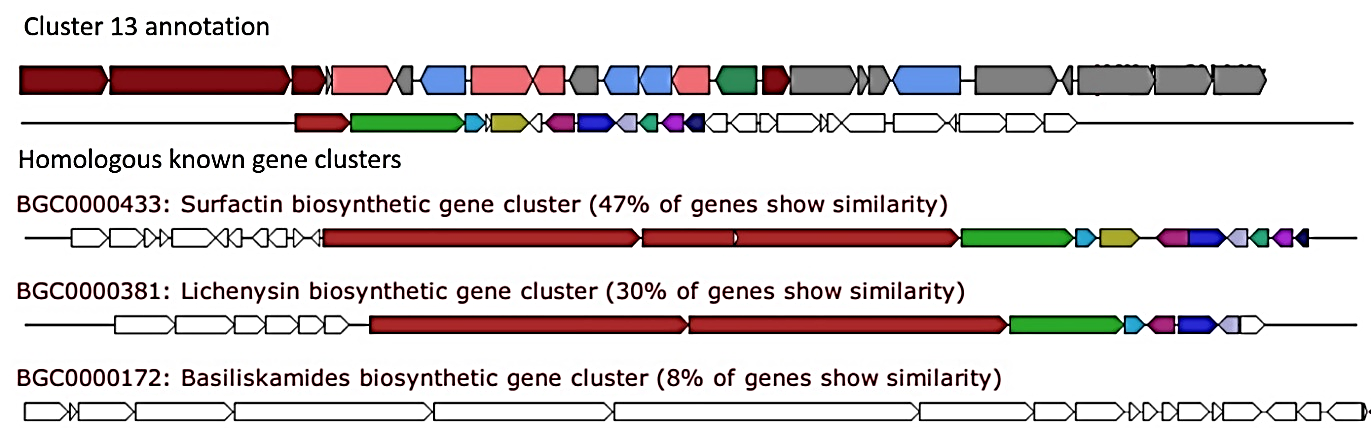


**Fig. S5d:** BGC predicted from cluster 13 of the BS10.5 genome includes surfactin (sfp gene) a major lipopeptide detected during amplification of PCR product of BS10.5 DNA extract.


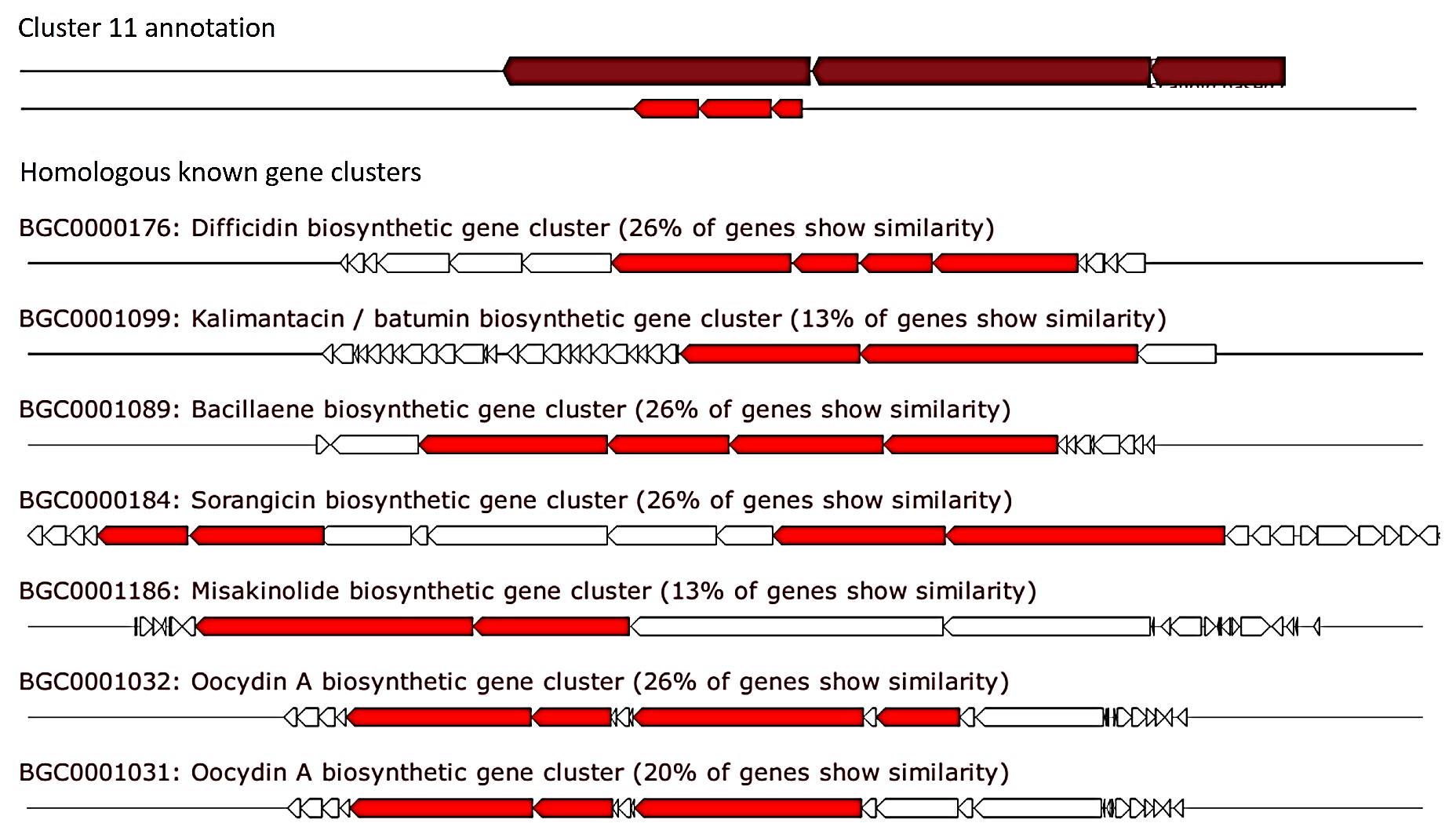


**Fig. S5e:** Major BGC predicted from the cluster 11 of the BS10.5 genome. The BGC showed similarity percentages lesser than 30.

**Fig. S5f:** Major BGC predicted from cluster 10 of the BS10.5 genome which had 2 different sub annotations. Fengycin was predicted at 13% similarity which was earlier detected during PCR-Gel electrophoresis.


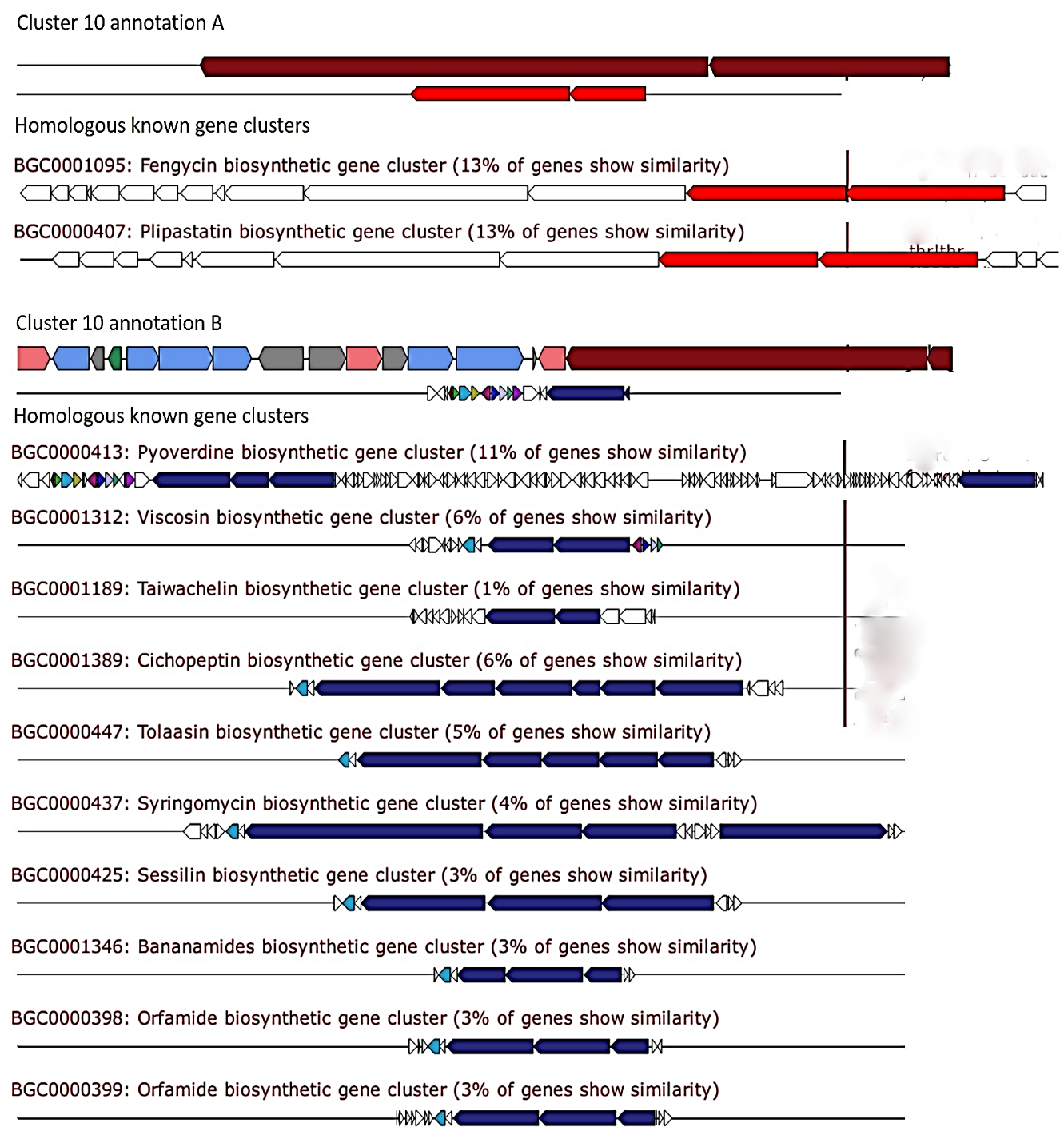


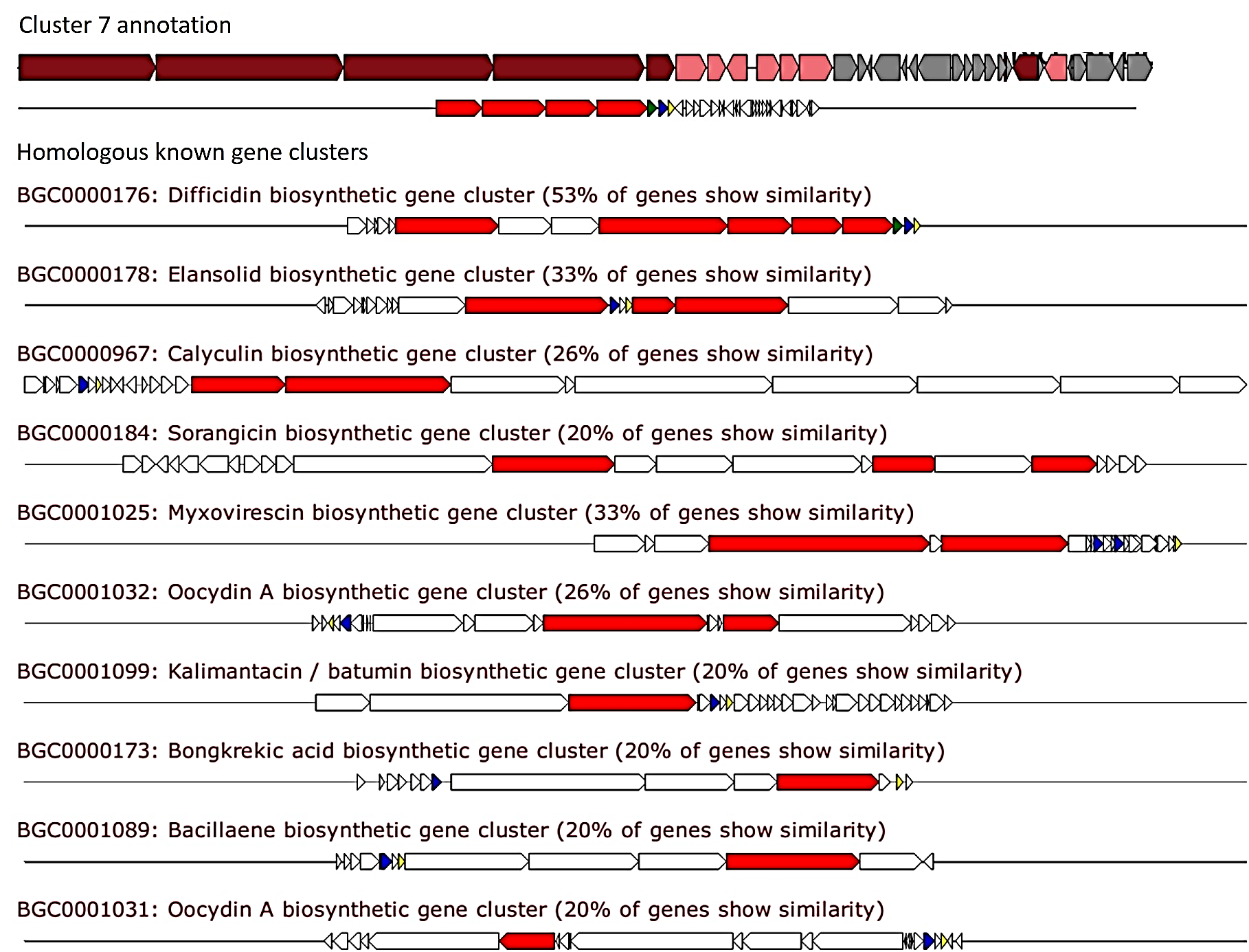


**Fig. S5g:** Major BGC predicted from cluster 7 of the BS10.5 genome


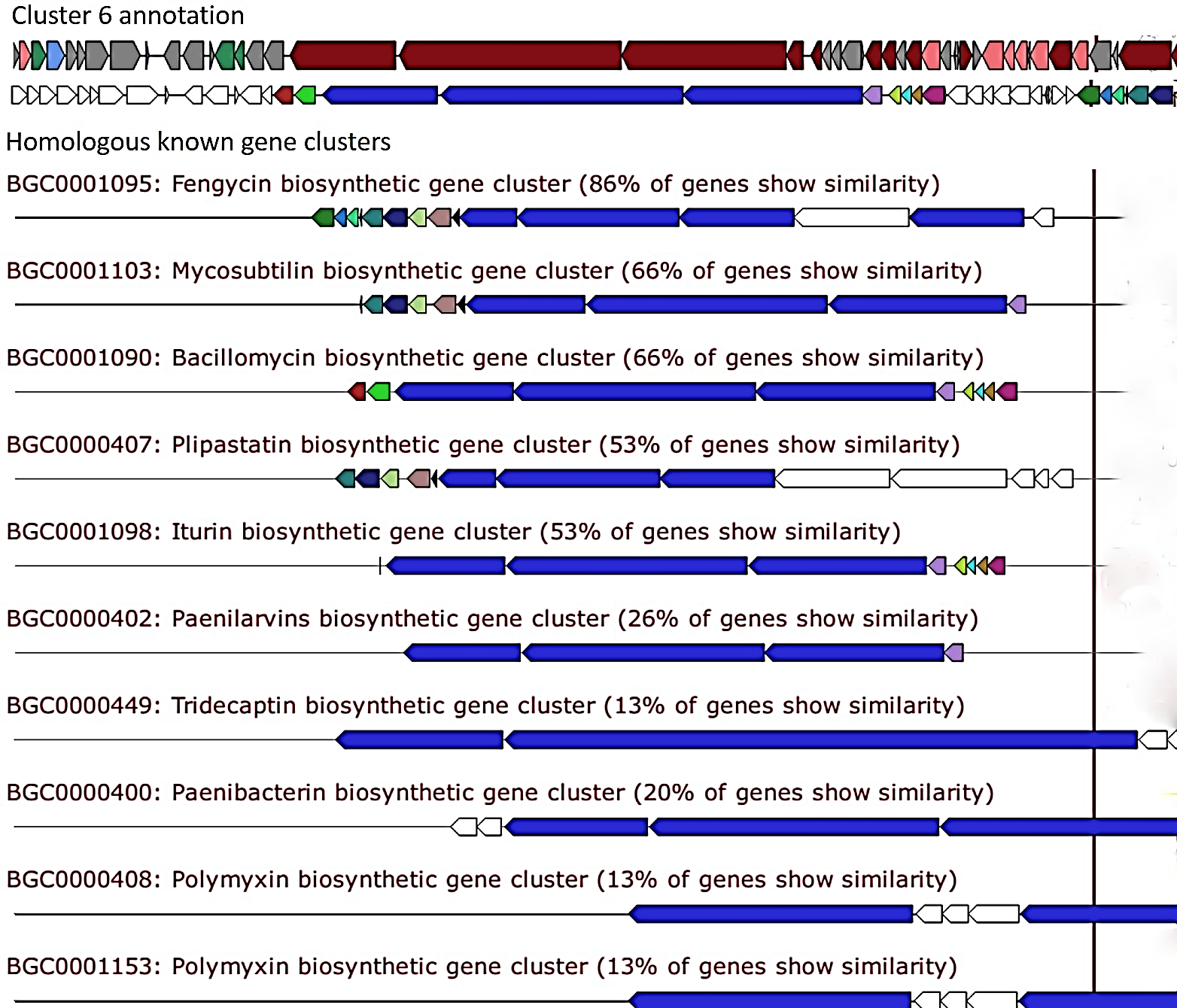


**Fig. S5h:** Major BGC predicted from cluster 6 of the BS10.5 genome.

**Fig. S5i:** Major BGC predicted from cluster 5 of the BS10.5 genome


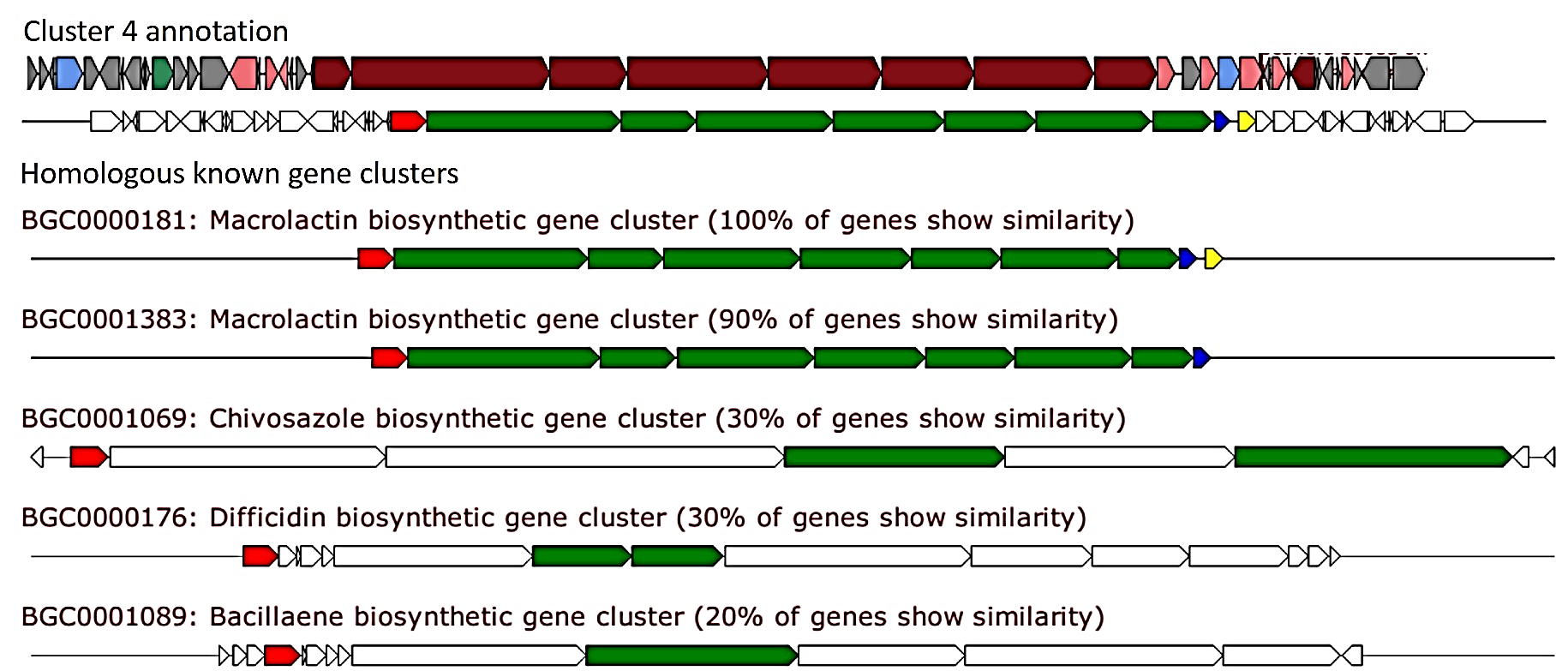

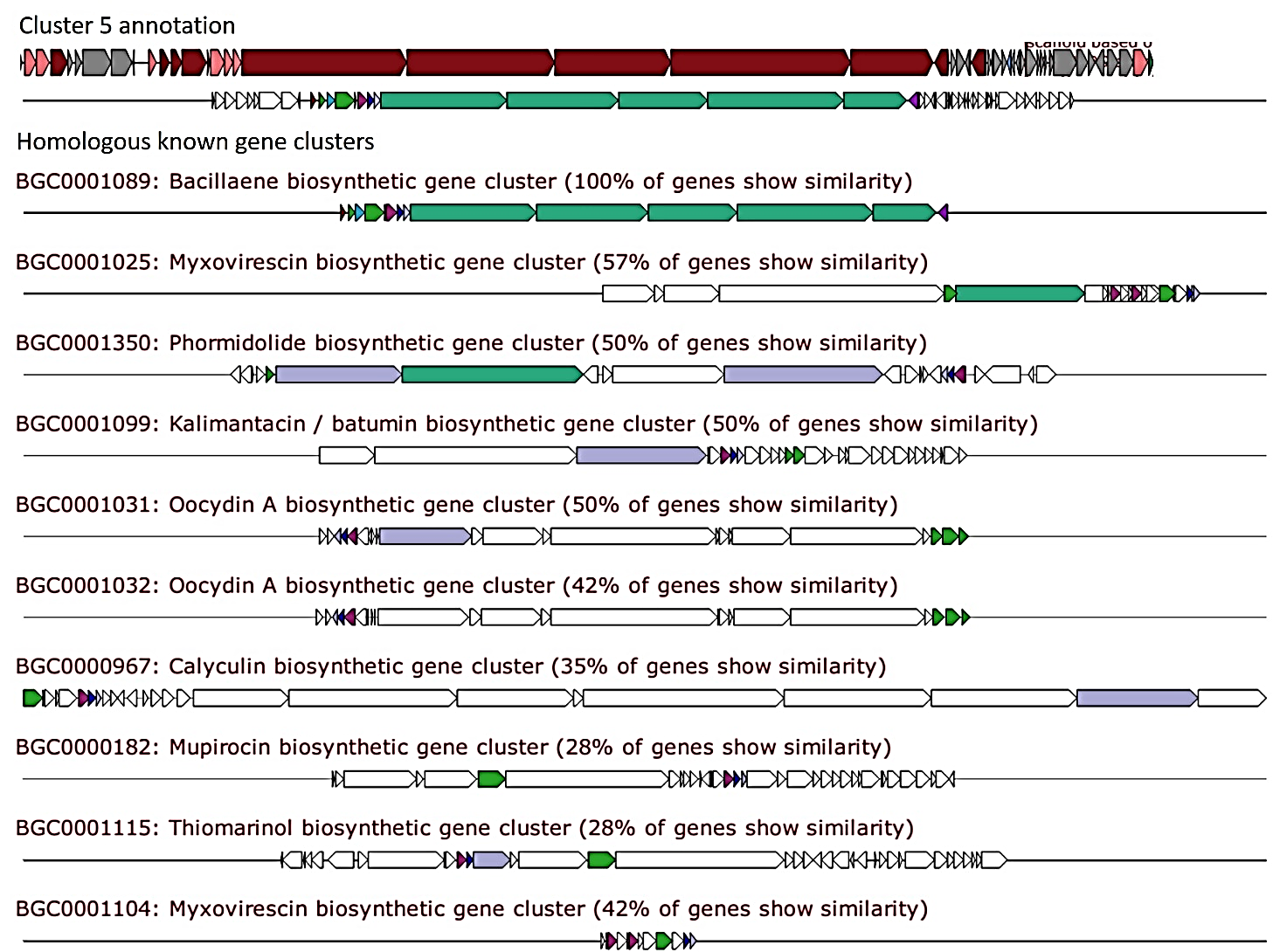


**Fig. S5j:** Major BGC predicted from cluster 4 of the BS10.5 genome


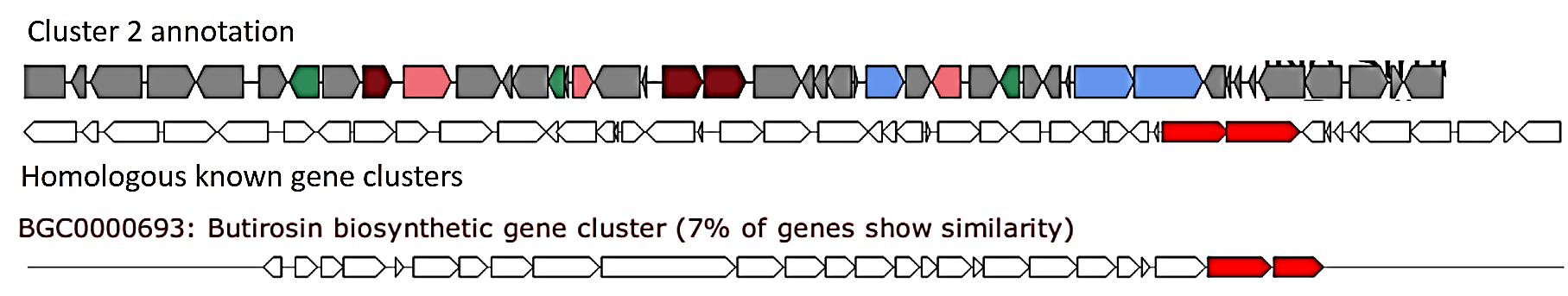


**Fig. S5k:** Major BGC predicted from cluster 2 of the BS10.5 genome


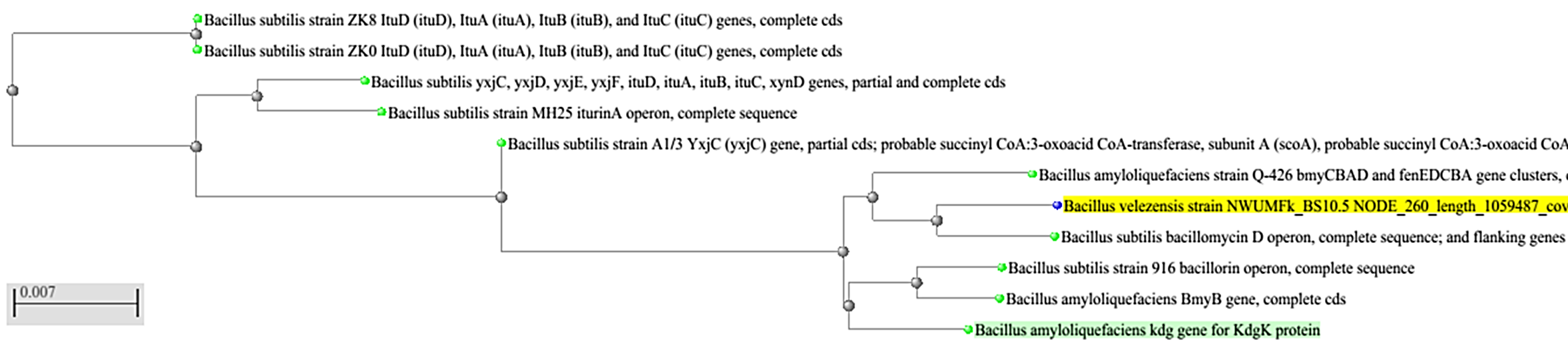


**Fig. S6:** Pangenomic tree of *B. veleznesis* strains. A node portion of the genome of BS10.5 clustered with other *Bacillus* strains that harbored gene required for the synthesis of known lipopeptide genes. The node_260_1059487 clustered directly with *B. subtilis* bacillomycin D operon and *B. amyloliquefaciens* strain Q-426 fengycin gene clusters. Strain *B. velezensis* NWUMFkBS10.5 is
